# Single-cell analyses of Crohn’s disease tissues reveal intestinal intraepithelial T cells heterogeneity and altered subset distributions

**DOI:** 10.1038/s41467-021-22164-6

**Published:** 2021-03-26

**Authors:** Natalia Jaeger, Ramya Gamini, Marina Cella, Jorge L. Schettini, Mattia Bugatti, Shanrong Zhao, Charles V. Rosadini, Ekaterina Esaulova, Blanda Di Luccia, Baylee Kinnett, William Vermi, Maxim N. Artyomov, Thomas A. Wynn, Ramnik J. Xavier, Scott A. Jelinsky, Marco Colonna

**Affiliations:** 1 Department of Pathology and Immunology, Washington University School of Medicine, St Louis, MO USA; 2 Pfizer Worldwide Research, Development and Medical, Cambridge, MA USA; 3 Department of Pathology, University of Brescia, Brescia, Italy; 4 Division of Gastroenterology, Washington University School of Medicine, St Louis, MO USA; 5 Immunology Program, Infectious Disease and Microbiome Program, Broad Institute of MIT, Cambridge, MA USA; 6 Present Address: Global Gene Therapy, Takeda Pharmaceuticals, Cambridge, MA USA; 7 Present Address: AbSci Inc, Vancouver, WA USA

**Keywords:** T cells, Mucosal immunology, Systems analysis, Inflammatory bowel disease

## Abstract

Crohn’s disease (CD) is a chronic transmural inflammation of intestinal segments caused by dysregulated interaction between microbiome and gut immune system. Here, we profile, via multiple single-cell technologies, T cells purified from the intestinal epithelium and lamina propria (LP) from terminal ileum resections of adult severe CD cases. We find that intraepithelial lymphocytes (IEL) contain several unique T cell subsets, including NKp30^+^γδT cells expressing RORγt and producing IL-26 upon NKp30 engagement. Further analyses comparing tissues from non-inflamed and inflamed regions of patients with CD versus healthy controls show increased activated T_H_17 but decreased CD8^+^T, γδT, T_FH_ and Treg cells in inflamed tissues. Similar analyses of LP find increased CD8^+^, as well as reduced CD4^+^T cells with an elevated T_H_17 over Treg/T_FH_ ratio. Our analyses of CD tissues thus suggest a potential link, pending additional validations, between transmural inflammation, reduced IEL γδT cells and altered spatial distribution of IEL and LP T cell subsets.

## Abstract (web summary)

Crohn’s disease results from transmural inflammation in the gut, but analyses of local immune populations are still lacking. Here, the authors show, by combining multiple single-cell approaches, that intraepithelial and lamina propria T cells are heterogenous, show unique phenotypes, and exhibit altered subsets upon inflammation.

## Introduction

Inflammatory bowel disease (IBD) encompasses intermittent chronic inflammatory disorders of the gastrointestinal tract that significantly impair the quality of life in affected individuals and can result in comorbidities and complications requiring repeated surgery^1,2^. IBD includes two major subtypes, Crohn’s disease (CD) and ulcerative colitis (UC). CD inflammation spans across all layers of the gut, while tissue damage in UC is confined to the mucosa. Both genetic and environmental factors contribute to IBD by generating abnormal interactions between the commensal microbiome and the mucosal immune system that result in uncontrolled intestinal inflammation. The conventional therapy with anti-inflammatory and immunomodulatory drugs has been recently integrated with biologicals that can effectively target cytokines, such as TNF-α, IL-12 and IL-23, or inflammatory cell recruitment with α4β7 blockers^3^. However, IBD still poses significant therapeutic challenges.

High-dimensional single-cell profiling approaches, such as single-cell RNA sequencing (scRNA-seq) and mass cytometry, have been recently performed on intestinal specimens from patients with CD or UC and controls. These studies provided unbiased analyses of cell lineages and their functional states in IBD, deconvoluted pathways underlying IBD pathogenesis and supplied biomarkers predicting the course of disease and the response to therapy^4–10^. While these studies have analyzed whole mucosal biopsies or the lamina propria (LP) selectively, few studies have analyzed intraepithelial lymphocytes (IEL) purified from CD specimens. IEL comprise a quite diverse and complex repertoire of TCRαβ^+^ and TCRγδ^+^ T cells^11,12^, which are strategically located at the interphase between the luminal environment and the intestinal barrier, contributing to intestinal homeostasis and mucosal protection^13,14^.

Here, we examine T cells from 90 intestinal specimens of CD and controls by either scRNA-seq, multi-parameter flow cytometry or CyTOF, with CD specimens derived from surgical resections of the terminal ileum of adult patients with severe CD. Comparing IEL T cell profiles with those of T cells purified from the LP, our data not only provide an unbiased view of T cell lineages diversity and functional states in the intestinal mucosa under both healthy and CD conditions, but also identify an altered spatial distribution of T cell subsets between the IEL and the LP compartments that potentially correlates with transmural inflammation, although this remains to be validated with larger patient cohorts.

## Results

### scRNA-seq analysis identifies multiple IEL T cell subsets

**Fig. 1:**
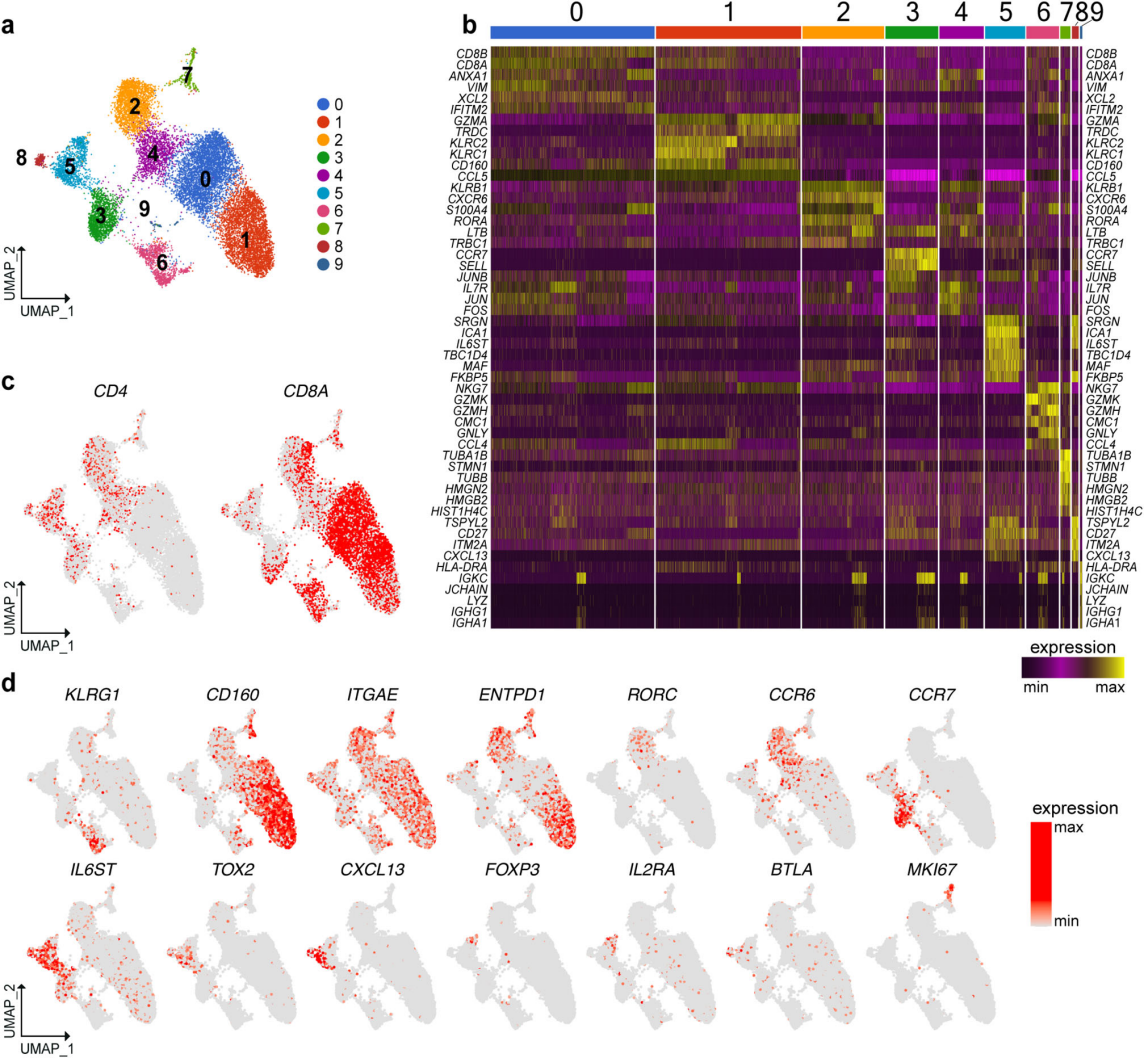
scRNA-seq of IEL T cells identifies discrete subsets of CD4^+^ and CD8^+^ T cells. **a** Unsupervised UMAP analysis of IEL T cell clusters. T cells were pooled from two controls and two CD patients (8822 cells control, 6909 cells CD). **b** Heat map displaying the top ten differentially expressed genes in each cell cluster. **c** Identification of *CD4* and *CD8A* expressing cells. **d** UMAP of representative selected genes associated with the identified clusters.

IEL were prepared from surgical resections of the terminal ileum of CD patients, which included both macroscopically inflamed tissue (II) and adjacent non-inflamed tissue (NI) (Supplementary Fig. 1a–c). Most CD cases required surgical treatment because of severity and resistance to medical therapy (patient information: Supplementary Data 1–3). Ileal resections from patients undergoing surgery for colonic polyposis or cancer were used as controls. In an initial survey, we defined the baseline heterogeneity of IEL T cells by scRNA-seq of about 15,000 cells sorted from two CD patients and two controls (sorting strategy Supplementary Fig. 1d). Unsupervised clustering by UMAP of gene expression data from both CD and control samples identified ten cell clusters (Fig. 1a, b). Differential expression of marker genes was used to annotate the different cell types and states. Three T cell clusters expressed *CD8A* (0, 1, and 6) and five expressed *CD4* (2, 3, 4, 5, and 8) (Fig. 1c). Among the CD8^+^ clusters, cluster 6 expressed genes indicating a canonical effector phenotype, including *KLRG1* (Fig. 1d)*, GZMB*, *GZMK*, *PRF1*, *IFNG,* and *FCRL6* (Supplementary Fig. 2a, b). Cluster 6 also expressed high level of *KLF2* (Supplementary Fig. 2c), a transcription factor that promotes lymphocytes circulation^15^. In contrast, cluster 1 and cluster 0 shared expression of *ITGAE*, the receptor for E-cadherin, which is indicative of tissue residency^16^, and *CD160*, a receptor for HVEM expressed on epithelial cells^17^ (Fig. 1d). Expression of *ENTPD1*, which encodes the activation marker CD39, distinguished cluster 1 from cluster 0 (Fig. 1d). The expression of γδTCR, NK cell receptors, cytotoxic mediators, and chemokines (Supplementary Fig. 2a, b) in a subset of cells within these two clusters suggested that additional subpopulations may be present (see below).

Within CD4^+^ cells, clusters 2 and 4 showed a T_H_17 transcriptional profile, featuring *RORC, CCR6* (Fig. 1d), *RORA, LTB, CCL20*, and *KLRB1* (Supplementary Fig. 2a–c). Both subsets expressed *CD40LG* (Supplementary Fig. 2b), which sustains T_H_17 differentiation in vitro and in vivo^18^. Moreover, cluster 2 expressed genes indicative of immediate effector function, including (a) cytokine genes *IL17A* and *IL26* (Supplementary Fig. 2a); (b) *IL23R*, which endows responsiveness to IL-23 (Supplementary Fig. 2b); (c) *ENTPD1* (Fig. 1d); (d) *CXCR6* (Supplementary Fig. 2b), which promotes retention in the intestinal epithelium^19^; (e) *CCL4* and *GZMB* (Supplementary Fig. 2a), known mediators of T_H_17 pathogenicity^20^. Cells in cluster 3 corresponded to naïve CD4^+^ T cells expressing *CCR7* (Fig. 1d)*, SELL, TCF7*, *LEF1*, as well as *KLF2* (Supplementary Fig. 2b, c). This cluster also contained a few naïve CD8^+^ T cells. Clusters 5 and 8 were enriched for genes related to T_FH_ and Treg lineages. These genes encoded cell surface receptors, such as *IL6ST* (for GP130) (Fig. 1d), *PDCD1* (for PD-1), *TIGIT*, *ICOS, TNFRSF4* (for OX40), and *IL6R* (Supplementary Fig. 2b), as well as transcription factors, such as *TOX2* (Fig. 1d), *MAF* and *BATF* (Supplementary Fig. 2c). The left portion of cluster 5 expressed *CXCL13* (Fig. 1d), *BCL6* and *CXCR5* (Supplementary Fig. 2b, c), pointing to a T_FH_ phenotype. The right portion of cluster 5 expressed the Treg markers *FOXP3*, *IL2RA, ENTPD1* (Fig. 1d), *CTLA4*, and *IL10* (Supplementary Fig. 2a, b). In addition, the Treg side of cluster 5 expressed *PRDM1* (Supplementary Fig. 2b), which facilitates Treg over T_FH_ differentiation^21^. Cluster 8 uniquely expressed *BTLA* (Fig. 1d) and *CD200* (Supplementary Fig. 2b), which are markers of terminal T_FH_ differentiation^22^. Cluster 7 included CD4 and CD8 *MKI67*^+^ proliferating cells (Fig. 1c, d), while cluster 9 (Fig. 1b) represented a small B cell contamination. Altogether, our survey of human IEL compartment shows the presence of circulating and resident CD8^+^ T cell subsets, as well as CD4^+^ T cell subsets belonging to the T_H_17, T_FH_, and Treg lineages.

### Flow cytometry of IEL corroborates T cell diversity

**Fig. 2:**
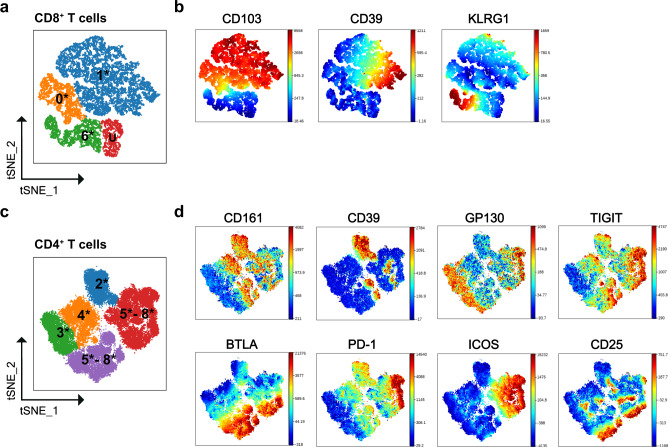
Identification of IEL CD8^+^ T cell and CD4^+^ T cell subsets by flow cytometry. **a** Schematic t-SNE clustering of IEL CD8^+^ T cells. Number illustrates the relationship with clusters observed by scRNA-seq. Asterisk illustrates the different experimental approach. U; undefined. **b** t-SNE clustering of IEL CD8^+^ T cells from a representative donor showing expression of CD103, CD39, and KLRG1. **c** Schematic t-SNE clustering of IEL CD4^+^ T cells. Number illustrates the relationship with clusters observed by scRNA-seq. Asterisk illustrates the different experimental approach. **d** t-SNE clustering of IEL CD4^+^ T cells from three representative donors showing expression of CD161, CD39, GP130, TIGIT, BTLA, PD-1, ICOS, and CD25.

To validate scRNA-seq data we analyzed IEL of CD and control patients by flow cytometry (gating strategy: Supplementary Fig. 3a, b**;** patient information Supplementary Data 4). Cells were clustered using viSNE analysis in Cytobank. To facilitate comparison of flow cytometry with scRNA-seq data, t-SNE clusters were labeled with the same numbers of the corresponding scRNA-seq cluster followed by an asterisk. We adopted CD103 as a marker of tissue-resident CD8^+^ T cells (Fig. 2a, b). Most CD103^–^ non-resident cells expressed KLRG1^+^ (cluster 6*) (Fig. 2b), consistent with the KLRG1^+^ T effector cells identified by scRNA-seq (cluster 6) (see Fig. 1d). CD103^+^ cells were split into CD39^+^ and CD39^–^ clusters (1* and 0*), which corresponded to scRNA-seq clusters 1 and 0. Flow cytometry also identified a unique cluster of CD103^–^KLRG1^–^ cells (U) that lacked all tested markers and thus could not match any subset identified by scRNA-seq (Fig. 2a).

Among CD4^+^ T cells, T_H_17 were marked by CD161 (Fig. 2c, d) and spanned clusters 2* and 4*, which were distinguished by expression of CD39 (Fig. 2d). Naïve CD4^+^ T cells (3*) encompassed GP130^+^TIGIT^–^BTLA^–^CD161^–^CCR6^–^CCR7^+/low^ cells (Fig. 2d). T_FH_ and Treg formed two large clusters (5–8*) which shared GP130, TIGIT, BTLA, and PD-1 as in scRNA-seq, but differed for ICOS expression (Fig. 2d). The external edges of clusters 5–8* expressed high level of CD25 (Fig. 2d) suggestive of fully differentiated Treg. Overall, flow cytometric data confirmed the presence of three distinct clusters of CD8^+^ T cells, as well as T_H_17, T_FH_, and Treg in the human IEL compartment.

### IEL show an aberrant representation of T cell subsets in CD

**Fig. 3:**
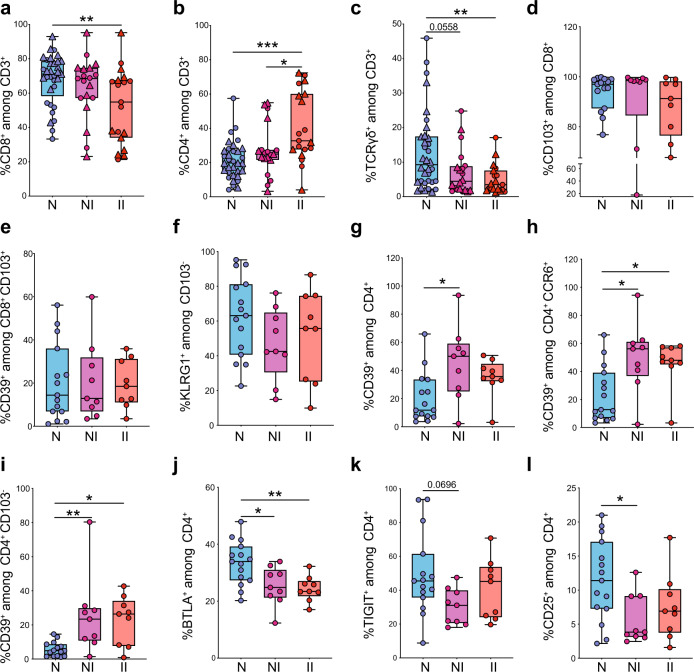
Quantification of IEL CD8^+^ T cell, CD4^+^ T cell subsets and γδ T cells by flow cytometry. **a** Percentages of IEL CD8^+^ among CD3^+^ T cells in terminal ileum of controls and CD patients. **b** Percentages of IEL CD4^+^ among CD3^+^ T cells in terminal ileum of controls and CD patients. **c** Percentages of IEL TCRγδ^+^ among CD3^+^ T cells in terminal ileum of controls and CD patients. **a**, **b** Controls (*N*), *n* = 33 (15 frozen, 18 fresh); CD, non-inflamed site (NI), *n* = 19 (9 frozen, 10 fresh); CD, inflamed site (II), *n* = 19 (9 frozen, 10 fresh). **c** Controls (*N*), *n* = 34 (16 frozen, 18 fresh); CD, non-inflamed site (NI), *n* = 20 (10 frozen, 10 fresh); CD, inflamed site (II), *n* = 19 (9 frozen, 10 fresh). Circles and triangles on the boxplots show data collected for each individual donor. Circles = frozen samples. Triangles = fresh samples. Data were median and interquartile range. **d**–**f** Quantification of total CD8^+^ T_RM_ (**d**), CD39^+^ T_RM_ (**e**), and KLRG1^+^CD103^–^ (**f**) T cells among IEL CD8^+^ from terminal ileum of controls and CD patients. **g**–**l** Percentages of CD39^+^ T cells (**g**), CD39^+^ CCR6^+^ (**h**), CD39^+^ CD103^–^ (**i**), BTLA^+^ (**j**), TIGIT^+^ (**k**), and CD25^hi^ (**l**) among IEL CD4^+^ from terminal ileum of controls and CD patients. **d**–**l** controls (*N*), *n* = 15; CD, non-inflamed site (NI), *n* = 9; CD, inflamed site (II), *n* = 9. Circles on the boxplots show data collected for each individual donor. Circles = frozen samples used in Fig. 3a–c. Data were median and interquartile range. Significance was calculated using an ordinary, one-way ANOVA, multiple comparisons test with Prism v8 software. **a** **P *=* 0.0094; **b** *P = 0.0178, ***P = 0.0001; **c** **P *=* 0.0081; **g** *P *=* 0.0148; **h** N vs. NI *P *=* 0.0134, N vs. II **P *=* 0.0310; **i** N vs. NI **P *=* 0.0079, N vs. II *P *=* 0.0214; **j** N vs. NI *P *=* 0.0204, N vs. II **P *=* 0.0087; **l** *P *=* 0.0315. Source data are provided as a Source Data file (Source Data 1).

To examine the impact of CD on IEL T cells, we quantified percentages of CD8^+^, CD4^+^, and γδ T cells in a large cohort of patients (Controls: *n* = 33–34; CD NI: *n* = 19–20; CD II: *n* = 19) by flow cytometry (Fig. 3a–c, Supplementary Data 5 and Source Data 1). CD8^+^ T cells were significantly decreased in CD patients at the inflamed site (Fig. 3a), a finding that was further supported by immunohistochemistry data (Supplementary Fig. 3c–f). CD8^+^ T cell attrition was paralleled by a significant increase in CD4^+^ T cells (Fig. 3b). In addition, γδ T cells were significantly reduced in the inflamed tissue of CD patients, as compared to controls (Fig. 3c). We further examined whether CD impacted specific T cell subsets in a fraction of the larger cohort of patients described above (Controls: *n* = 15; CD NI *n* = 9; CD II *n* = 9). Quantification of resident CD103^+^ CD8^+^ T cells and non-resident CD103^–^KLRG1^+^ CD8^+^ T cells did not reveal major differences between CD and controls (Fig. 3d–f), indicating global rather than subset-specific reduction of CD8^+^ T cells in CD. In contrast, quantification of different CD4^+^ T cell clusters showed a significant increase of CD39^+^CD4^+^ T cells, CD39^+^CCR6^+^ T_H_17 cells (Fig. 3g, h) and CD39^+^CD4^+^ T_H_17 within the non-resident CD103^–^ population (Fig. 3i) in CD patients, suggesting that these cells may have recently settled into the tissue. In addition, BTLA^+^ and TIGIT^+^ CD4^+^ T cells were decreased in CD patients (Fig. 3j, k), indicating reduced representation of T_FH_. Finally, we observed a significant decrease in the relative proportion of CD25^hi^ CD4^+^ T cells in CD, which may correspond to Tregs (Fig. 3l). Altogether, these results suggested that the IEL compartment of CD patients exhibits an aberrant T cell landscape. Notably, some CD-associated alterations were evident in the non-inflamed tissue. The non-inflamed tissue may exhibit early cellular changes reflecting ongoing disease without any macroscopic sign of inflammation and may predict risk of recurring inflammation.

### Reclustering reveals T cell subsets with unique features

**Fig. 4:**
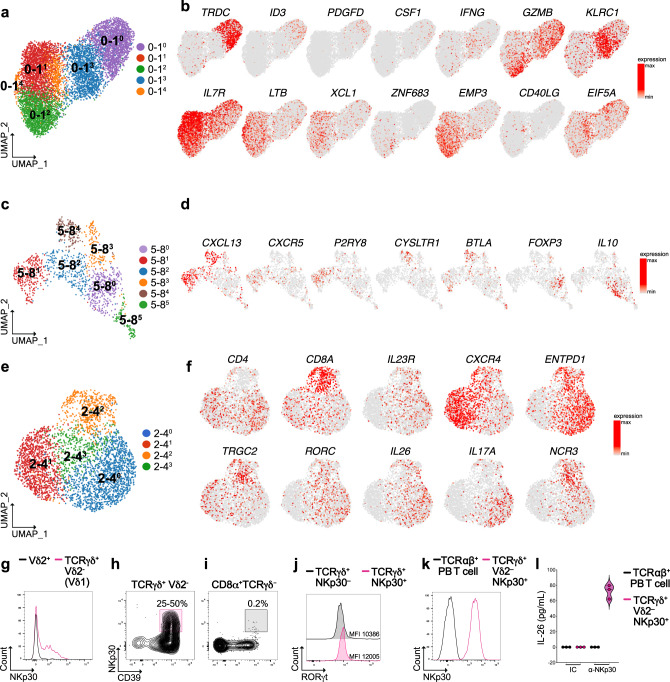
Reclustering of heterogenous IEL CD8^+^ and CD4^+^ T cells populations. **a** Unsupervised UMAP reclustering of clusters 0 and 1 from Fig. 1a. **b** UMAP of representative selected genes associated with the identified clusters. **c** Unsupervised UMAP reclustering of clusters 5 and 8 from Fig. 1a. **d** UMAP of representative selected genes associated with the identified clusters. **e** Unsupervised UMAP reclustering of clusters 2 and 4 from Fig. 1a. **f** UMAP of representative selected genes associated with the identified clusters. **g** Overlay expression of NKp30 in Vδ2^–^ (pink) vs. Vδ2^+^ (black) γδ T cells. **h**, **i** Representative flow plots showing the expression of NKp30 in TCRγδ^+^Vδ2^–^ CD39^+^ (**h**) and CD8^+^TCRγδ^–^CD39^+^ (**i**). T cells in terminal ileum of control patients (one donor representative of nine is shown). **j** Overlay expression of RORγt in NKp30^–^ (black) vs. NKp30^+^ γδ T cells (pink). **k** Overlay expression of NKp30 in peripheral blood TCR αβ^+^ T cells (black) vs. NKp30^+^ sorted γδ T cells (pink). **l** IL-26 production by NKp30^+^ γδ T cells upon antibody mediated crosslinking of NKp30. MFI mean fluorescence intensity, PB peripheral blood, IC isotype control.

To further dissect T cell heterogeneity within the major IEL populations, we reclustered scRNA-seq data of three pairs of clusters (see Fig. 1a). These included tissue-resident CD8^+^ T cell clusters 0 and 1 (0–1), the T_FH_-Treg clusters 5 and 8 (5–8), and the T_H_17 clusters 2 and 4 (2–4). Subclusters were indicated with a superscript number next to the original cluster pair. The 0–1 cluster pair was resolved into five specialized subsets (Fig. 4a). Cluster 0–1^0^ corresponded to γδ T cells, as indicated by the expression of transcripts for γδTCR (*TRDC* and *TRGC1*), as well as the transcription factors *ID3*^23^ and *HOPX* (Fig. 4b and Supplementary Fig. 4a). These γδ T cells selectively expressed the signaling adapter *SH2D1B* as well as *PDGFD* and *CSF1*. Cluster 0–1^3^ encompassed a CD8^+^ T cell subset that stood out for production of IFN-γ (*IFNG*), granzyme B (*GZMB*), and expression of the NK cell inhibitory receptor *KLRC1* (Fig. 4b and Supplementary Fig. 4a). Both clusters 0–1^0^ and 0–1^3^ shared expression of CD39 (*ENTPD1*) and several NK cell receptors, including *KIR2DL4*, *KLRC2*, *KLRD1,* and *SLAMF7*. Cluster 0–1^1^ expressed *IL7R*, *IL2*, and *TCF7* (Fig. 4b and Supplementary Fig. 4a), which are indicative of long-lived and memory-like properties^24,25^, as well as lymphotoxin B (*LTB*), and the DC attracting chemokines *XCL1* and *XCL2*^26^. Cluster 0–1^2^ expressed the master transcription factor for tissue residency *ZNF683*^27^, and markers of cytotoxicity, such as *GZMB*, *PRF1*, and *TNFSF10* (Fig. 4b and Supplementary Fig. 4a). As opposed to clusters 0–1^0^ and 0–1^3^, this cluster did not exhibit CD39 or NK cell receptors but expressed the epithelial membrane protein 3 (*EMP3*) (Fig. 4b and Supplementary Fig. 4a). The small cluster 0–1^4^ contained CD8^+^ T cells marked by *CD40LG* and a network of p53 regulators, such as *EIF5A, MDM4*, and *SET*^28^ (Fig. 4b and Supplementary Fig. 4a). CD8^+^ T cell clusters 0–1^1^, 0–1^4^, and 0–1^2^ produced the *S100A* family members *A4*, *A6*, and *A10*, which have antimicrobial functions (Supplementary Fig. 4a).

We also re-analyzed the cluster pair 5 and 8 including T_FH_ and Treg. This analysis identified six clusters (Fig. 4c). Clusters 5–8^1^ and 5–8^4^ represented two distinct clusters of T_FH_, both of which expressed the chemokine *CXCL13* and the T_FH_ master transcription factor *BCL6* (Fig. 4d and Supplementary Fig. 4b). However, 5–8^1^ uniquely expressed *CXCR5*, *TNFSF8*, and the G-protein-coupled receptor *P2RY8*^29^. Conversely, cluster 5–8^4^ expressed *CYSLTR1*^30^ (Fig. 4d and Supplementary Fig. 4b). Cluster 5–8^4^ also expressed *BTLA*^29^, as well as unique genes, such as amyloid precursor protein (*APP*), *MS4A6A*, and *SERPINE2* (Supplementary Fig. 4b). Cluster 5–8^0^ encompassed Treg and contained both *FOXP3*^+^ Treg (right side), as well as *LAG3*^+^ Tr1 producing IL-10 (left side)^30^. Cluster 5–8^2^ was located between Treg and T_FH_, shared expression of *PRDM1* with Treg, but showed residual expression of *CXCR5* and CX*CL13*; this subset may reflect a T_FH_-Treg plasticity reported both in human and mouse^31^. Cluster 5–8^3^ encompassed cells expressing the γδTCR transcripts *TRCG1* and *TRCG2*, as well as *CD8A*, *CCL5*, and the cytotoxic mediators *GZMA* and *GZMB* (Supplementary Fig. 4b). These cells may be γδ T cells or CD8^+^ αβ T cells expressing *TRCG1* and *TRCG2* transcripts^32^. Noteworthy, a few cells in this cluster expressed *FOXP3*. Because of a lack of specific markers, cluster 5–8^5^ could not be explicitly identified.

We finally re-analyzed the CD4^+^ T cell cluster pair 2–4, because it contained a small group of *CD8A*^+^ cells among a large population of T_H_17 cells (see Fig. 1c). Reclustering of the 2–4 cluster pair generated four distinct subsets (Fig. 4e). Cluster 2–4^0^ corresponded to activated cytokine-secreting *IL23R*^+^ T_H_17, whereas cluster 2–4^1^ expressed *CXCR4*, *GPR183*, and *CXCR3*, which indicate quiescent T_H_17^33^ (Fig. 4f and Supplementary Fig. 4c). Cluster 2–4^2^ belonged to the γδ T cell lineage, as indicated by *TRCG2* and *CD8A* expression. These cells expressed *RORC*, *IL17A*, and *IL26*, explaining the initial co-clustering with T_H_17 cells and suggesting that they may represent a discrete subpopulation of γδ T cells with a type 17 polarization (Fig. 4f). This γδ T cell subset was also marked by *NCR3* (Fig. 4f). To validate this γδ T cell subset, we stained IEL with a mAb for TCRVδ2, followed by an antibody that recognizes all γδ TCRs, and a mAb for NKp30 (patient information in Supplementary Data 6). Among Vδ2^–^ γδ^+^ T cells, about 25–50% of the cells expressed NKp30 in different individuals (*n* = 9), suggesting that NKp30^+^ γδ T cells represent approximately half of the TCRVδ2^–^Vδ1^+^ γδ T cells (Fig. 4g, h and Supplementary Fig. 4d). All of these cells expressed CD39 (Fig. 4h). In contrast, NKp30 was not expressed by terminal ileum CD8^+^TCRγδ^–^ T cells (Fig. 4i and Supplementary Fig. 4d), contrary to what shown for colonic CD8^+^ T cells of UC patients^10^.

To validate the functional relevance of NKp30 on this unique γδ T cell subset, we sorted all γδ T cells from control patients and tested the expression of RORγt on NKp30^+^ vs. NKp30^–^ cells. As shown in Fig. 4j, NKp30^+^ γδ T cells expressed higher level of RORγt than NKp30^–^. We further expanded NKp30^+^ γδ T cells in vitro to perform functional assays. Expanded γδ T cells maintained high levels of NKp30 expression (Fig. 4k) and produced IL-26 upon engagement of NKp30 with a cognate antibody (Fig. 4l). Because of the reported antibacterial properties of IL-26^34^, our results suggest that this discrete γδ T cell subset may have a protective role in intestinal homeostasis.

### T cell clusters in LP are differentially activated and represented compared to IEL

**Fig. 5:**
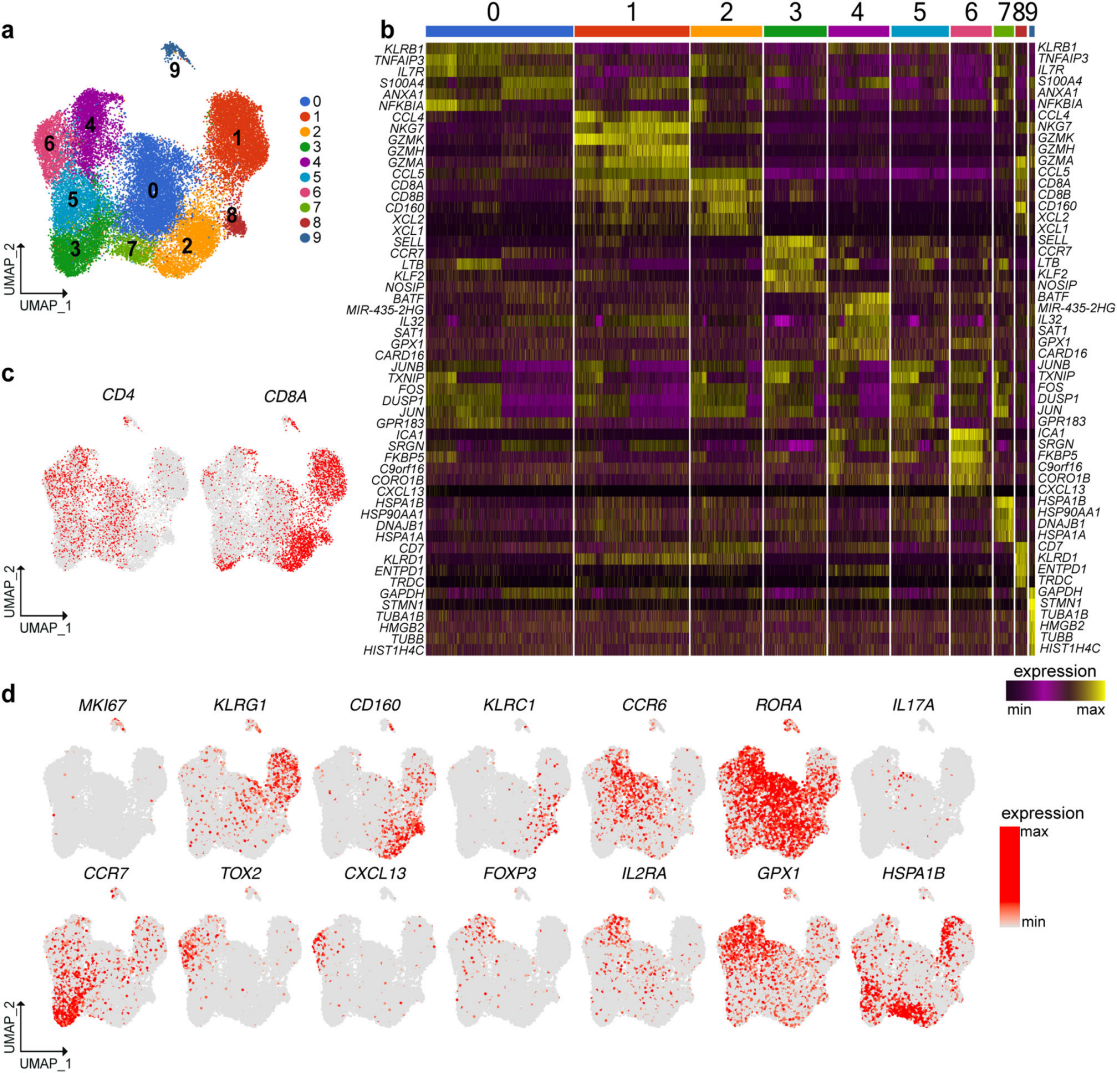
scRNA-seq of LP T cells identifies discrete subsets of CD4^+^ and CD8^+^ T cells. **a** Unsupervised UMAP analysis of LP T cell clusters. T cells were pooled from two controls and two CD patients (9107 cells control, 20,140 cells CD). **b** Heat map displaying the top ten differentially expressed genes in each cell cluster. **c** Identification of *CD4* and *CD8A* expressing cells. **d** UMAP of representative selected genes associated with the identified clusters.

We next surveyed the baseline heterogeneity of CD3^+^ cells in the LP by scRNA-seq of 29,247 cells sorted from the same CD and control samples analyzed for IEL (Supplementary Fig. 1a–c, e; patient information in Supplementary Data 1). Unsupervised clustering of scRNA-seq data from LP T cells by UMAP identified nine clusters (Fig. 5a, b). Three T cell clusters expressed *CD8A* (1, 2, and 8), six expressed *CD4* (0, 3, 4, 5, 6, and 7) (Fig. 5c), while one (9) expressed both and was enriched in cell cycle genes, such as *MKI67, STMN1, TUBB*, and *TUBA1B* (Fig. 5b, d). Within *CD8A*^+^ clusters, cluster 1 showed the hallmarks of effector CD8^+^ T cells: *KLRG1* (Fig. 5d)*, EOMES, TBX21*, *CCL4, CCL3, CCL5, IFNG, PRF1, GZMB, GZMA, GZMH*, and *GZMK* (Supplementary Fig. 5a, b). This cluster was reminiscent of IEL cluster 6. Cluster 2 and 8 expressed the HVEM receptor *CD160* and *ITGA1* (Fig. 5d and Supplementary Fig. 5c), and the chemokines *XCL1 and XCL2* (Supplementary Fig. 5b). Cluster 8 expressed the NK cell receptors *KLRC1*, *KIR2DL4*, *KLRC2, KLRD1*, *TRDC*, and *ENTPD1* (Fig. 5b, d and Supplementary Fig. 5b). Overall, LP clusters 2 and 8 paralleled IEL clusters 0 and 1, respectively.

Among CD4 T cells, cluster 0 corresponded to T_H_17 based on expression of *CCR6, RORA* (Fig. 5d), *KLRB1, LTB*, and *CCL20* (Supplementary Fig. 5b, c). Only few cells on the top of the cluster were enriched in genes indicative of effector function, such as *IL17A*, *IL23R*, *IL26*, *RORC*, *CXCR6*, *GZMB*, *ENTPD1*, and *CCL4* (Fig. 5d and Supplementary Fig. 5a–c), indicating that LP contains more quiescent than activated T_H_17 compared to IEL (Supplementary Fig. 6a). Notably, cluster 0 was also enriched in *LGALS3* and *GPR65* (Supplementary Fig. 5c)*. LGALS3* encodes galectin 3 that has antibacterial and antifungal immunity^35^, corroborating antimicrobial properties of T_H_17^36^. *GPR65* is a proton sensing G-protein-coupled receptor that has been found to be a risk factor for IBD^37^. Cluster 3 corresponded to naïve T cells expressing *CCR7* (Fig. 5d)*, LEF1, TCF7, SELL*, and *KLF2* (Supplementary Fig. 5a, c). Cluster 5 and 6 were both T_FH_ based on *TOX2* and *CXCR5* expression (Fig. 5d and Supplementary Fig. 5c); cluster 6 was further distinguishable from cluster 5 based on the selective expression of *CXCL13* (Fig. 5d), *PDCD1*, *BTLA*, and *CD200* (Supplementary Fig. 5c). As opposed to IEL T_FH_, LP T_FH_ were not readily distinguishable into *P2RY8*^+^ and C*YSLTR1*^+^ subsets. Cluster 4 corresponded to Treg expressing *FOXP3, IL2RA* (Fig. 5d), *ENTPD1*, *BATF, IL10, LAIR2, TNFRSF4* (for OX40), and *TNFRSF9* (for CD137) (Supplementary Fig. 5a–c). The Treg subset was enriched in *GPX1* (Fig. 5d) and *GLRX* (Supplementary Fig. 5b), which are induced by FOXP3 and encode molecules protecting from oxidative stress^38,39^. Cluster 5, 6, and 4 shared expression of *CTLA4*, *TIGIT*, *ICOS* (Supplementary Fig. 5c) and the transcription factors *TOX*, *TOX2*, and *MAF* (Fig. 5d and Supplementary Fig. 5a), corroborating that T_FH_ and Treg lineages are closely related^31^. Finally, cluster 7 identified a unique cluster of CD4^+^ T cells exhibiting a heat-shock stress-activated pathway, as indicated by expression of HSP family members (*HSPA1A, HSPA1B*, and *DNAJB1*)*, JUN*, *TNF*, and *IL2* (Fig. 5b, d and Supplementary Fig. 5a, b). The activation of this pathway was also evident at the bottom of the *CD8A*^+^ cluster 2 (Fig. 5c, d). Altogether, our data indicated that IEL and LP harbor overlapping T cell subsets, although T_H_17 in LP are more quiescent than those in the IEL compartment (Supplementary Fig. 6a). Furthermore, LP contained a unique subset of T cells that express heat-shock stress-pathway genes and secreted cytokines such as *TNF* and *IL2*.

### Severe CD modifies the T cell landscape in the LP

**Fig. 6:**
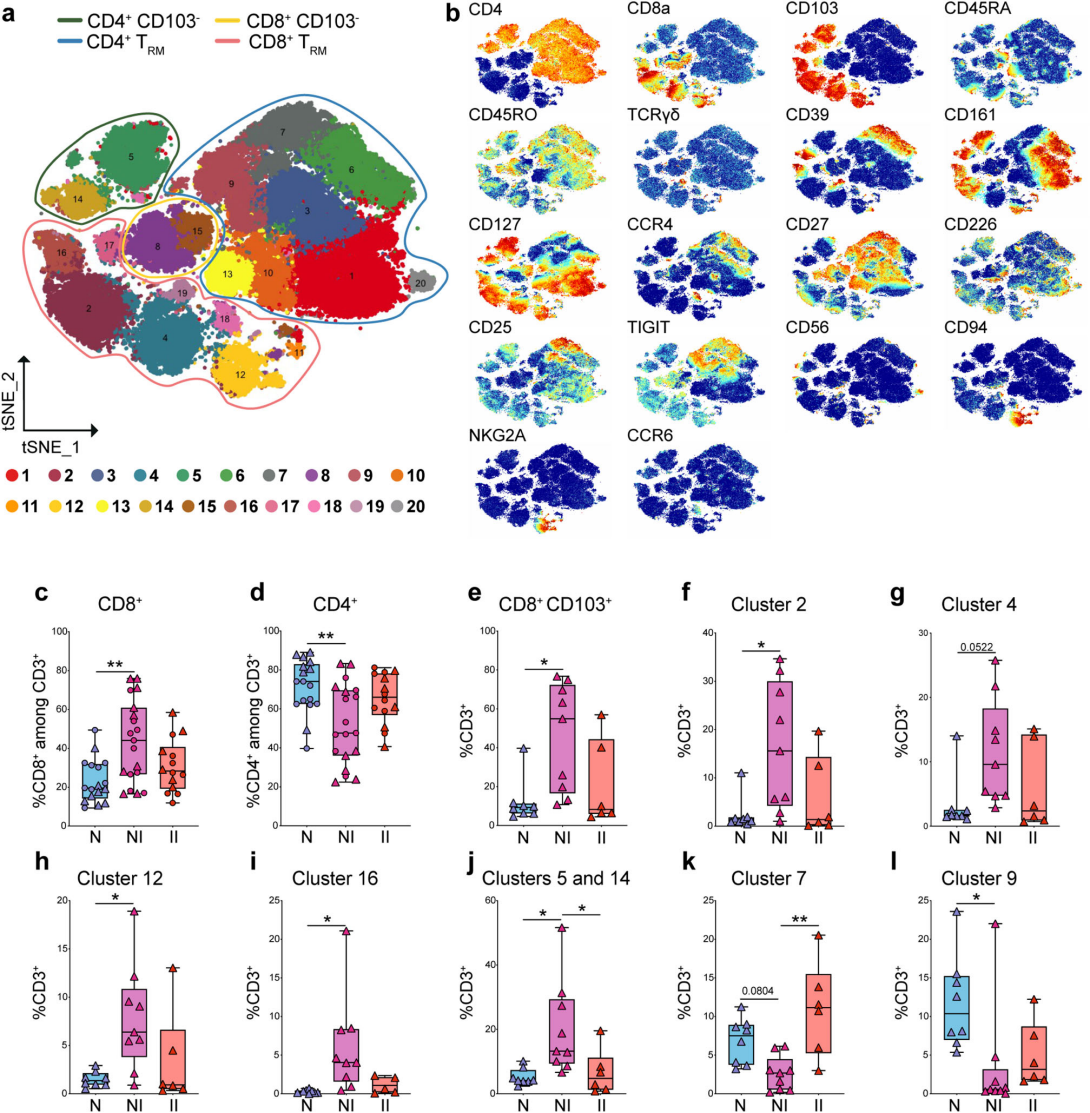
Quantification of CD8 and CD4 T cell clusters by CyTOF analysis in LP of control and CD patients. **a** Schematic t-SNE of CD4^+^ and CD8^+^ T cells from LP of all donors concatenated together (*n* = 18) controls (*N*), *n* = 8; CD, non-inflamed site (NI), *n* = 9; CD, inflamed site (II), *n* = 6. Total of 23 samples. **b** t-SNE of the indicated markers in CD4^+^ and CD8^+^ T cells. **c**, **d** Quantification of total CD8^+^ (**c**), total CD4^+^ (**d**) in LP of controls and CD patients by CyTOF (triangles = fresh samples) and FACS (circles = frozen samples). **c**, **d** Control (*N*), *n* = 17 (8 fresh, 9 frozen); CD, non-inflamed site (NI) *n* = 19 (9 fresh, 10 frozen); CD, inflamed site (II), *n* = 14 (6 fresh, 8 frozen). **e**–**i** Quantification of total CD8^+^ T_RM_ (**e**) and CD8^+^ clusters 2 (**f**), 4 (**g**), 12 (**h**), and 16 (**i**) in LP of controls and CD patients by CyTOF. **j**–**l** Quantification of the CD4^+^ clusters 5 and 14 (**j**), 7 (**k**), and 9 (**l**) in LP of controls and CD patients by CyTOF. **e**–**l** Controls (*N*), *n* = 8; CD, non-inflamed site (NI), *n* = 9; CD, inflamed site (II), *n* = 6. Circles and triangles on the boxplots show data collected for each individual donor. Data were median and interquartile range. Significance was calculated using an ordinary, one-way ANOVA, multiple comparisons test with Prism v8 software. **c** **P *=* 0.0014; **d** **P *=* 0.028; **e** *P *=* 0.0139; **f** *P *=* 0.0178; **h** *P *=* 0.0178; **i** *P *=* 0.0219; **j** N vs. NI *P *=* 0.0156, NI vs. II *P *=* 0.0465; **k** **P *=* 0.0014; **l** **P *=* 0.0283. T_RM_ tissue-resident memory T cell. Source data are provided as a Source Data file (Source Data 2).

To validate scRNA-seq analysis of LP and quantify differences related to disease status, we performed mass cytometry on fresh LP T cells of CD (NI: *n* = 9; II *n* = 6) and control patients (*n* = 8) (patient information: Supplementary Data 7). Dimensionality reduction by viSNE analysis enabled to solve T cell heterogeneity in 20 different clusters. Notably, mass cytometry identified more clusters than scRNA-seq analysis, suggesting that this technique may better distinguish cell subsets that express shared markers at different levels. Eight clusters expressed CD8 (15, 8, 17, 16, 2, 4, 18, and 12), ten clusters expressed CD4 (14, 5, 13, 10, 9, 7, 6, 3, 1, and 20), a small cluster of γδ T cells (19) expressed neither CD4 or CD8, and a tiny cluster (11) remained undefined (Fig. 6a, b). CD103 distinguished tissue-resident CD8^+^ and CD4^+^ T cells from circulating cells. CD103^–^ non-resident CD8^+^ T cells included clusters 15 and 8, which corresponded to naïve CD45RA^hi^ and memory CD45RO^+^ CD8^+^ T cells, respectively. CD103^+^ resident CD8^+^ T cell clusters 18, 2, 4, and 12 were separated based on expression of CD161 (16 and 2), CD39 (16), CD94 and NKG2A (12), and CCR4 (18) (Fig. 6a, b). Cluster 17, which mapped close to clusters 15 and 8 between circulating and tissue-resident cells, exhibited low/intermediate levels of CD103 (Fig. 6b), suggesting that it may represent recently immigrated CD8^+^ T cells settling into the tissue.

Within CD4^+^ T cells, CD103^–^ clusters 1, 3, 6, and 20, corresponded to circulating memory T_H_17 cells expressing CD161, CD45RO, and CD127 (Fig. 6a, b). The four clusters were distinguished based on the expression of CD39, CCR4 (6), CD27 (6 and 3), and CD226 (1 and 20) (Fig. 6a, b). The expression of CD226 may be suggestive of pathogenicity, as CD226 has been implicated in autoimmunity^40^. Clusters 5 and 14 represented tissue-resident memory T_H_17 because of the expression of CD103, CD45RO, and CD161. As in IEL, these cells were split in CD39^+^ and CD39^–^ subsets. Cluster 7 consisted of Treg as indicated by high CD25 and CD39 expression (Fig. 6b), while cluster 9 included T_FH_ cells based on high TIGIT expression^41^ (Fig. 6b). Clusters 13 represented naïve CD45RA^hi^ CD45RO^–^ CD4^+^ T cells, while cluster 10 was marked by expression of CD45RO and loss of CD45RA, indicative of a memory phenotype (Fig. 6a, b). Finally, cluster 20 was distinguished based on CD56 expression (Fig. 6b), which is typical of cytokine-induced killer cells.

Quantification of different LP clusters in CD and control patients revealed an opposite trend than what observed in IEL, with a significant increase in CD8^+^ T cells paralleled by a decrease in CD4^+^ T cells (Fig. 6c, d; fresh samples triangles). These data were confirmed by flow cytometry in a different cohort of frozen samples from control (*n* = 9) and CD (*n* = 11) patients and combined to the CyTOF data (Fig. 6c, d**;** frozen samples circles; patient information: Supplementary Data 7 and Source Data 2). Among CD8^+^ T cells, CD103^+^ resident cells were increased in CD compared to controls (Fig. 6e), as evidenced by expansion of clusters 2, 4, 12, and 16 (Fig. 6f–i). Although total CD4^+^ T cells were reduced in CD, T_H_17 of clusters 5 and 14 were significantly increased at the non-inflamed site of CD lesions (Fig. 6j). On the contrary, Treg (7) and T_FH_ (9) were decreased (Fig. 6k, l). The increase in T_H_17 cells (CD4^+^CCR6^+^CD161^+^) and decrease of T_FH_ (TIGIT^+^) was further confirmed by running CITRUS analysis (Supplementary Fig. 7a–c). Taken together, our data suggested that inflammatory T_H_17 and CD8^+^ T cells are increased in the LP of CD, paralleled by an attrition of CD4^+^ T cells with regulatory properties, consistent with deepened gut wall inflammation.

## Discussion

Through a high-resolution analysis of human intestinal IEL T cells in controls and severe adult CD, our study defined a vast heterogeneity of T cell lineages in the IEL compartment, including various subsets of CD8^+^, γδ^+^, and CD4^+^ T cells. Compared to controls, CD was associated with major abnormalities in the composition of IEL T cells, which included: (a) an increase in inflammatory CD39^+^ T_H_17; (b) a decrease in Treg, which might exacerbate inflammation; (c) a decrease in T_FH_, which may explain the impaired mucosal IgA production previously reported in CD^42^; and (d) a global reduction of CD8^+^ T cells and γδ T cells. These changes in the IEL compartment were coupled with increased CD8^+^ T cells and T_H_17, as well as reduced T_FH_ and Treg in the LP, likely reflecting the deepening of inflammation and overt transmural damage^1,2^. Overall, despite some limitations of our study due to cohort heterogeneity of CD patients, our results offer insight into T cell correlates of transmural inflammation and relapsing/recurrent disease.

The remarkable heterogeneity of human T cells identified in the IEL compartment reflected the presence of different T cell lineages, as well as various stages of differentiation, activation, and tissue residency within each lineage. T_H_17 cells included quiescent cells and cells capable of immediate effector functions, which were readily distinguished based on the mutually exclusive expression of *CXCR4* and *CD39*, among other distinctive markers. IEL CD39^+^ T_H_17 may be a two-edged sword. On one hand, they exhibit pathogenic features, such as expression of *GZMB* and *CCL4*, suggesting that they may be cytotoxic against epithelial cells and recruit inflammatory cell types that promote tissue destruction. On the other hand, production of IL-17 and IL-26 may enhance barrier function, providing protection. Given that CD39 has been shown to sustain T_FH_ survival by degrading proapoptotic ATP released in the intestinal environment^43^, CD39 may sustain survival of the T_H_17 cells. Further increasing diversity, T_H_17 expressed different levels of *CD103*, indicating the presence of T_H_17 at different stages of migration ranging from circulating to tissue-resident.

T_FH_ also included two major subsets: one subset expressed *P2RY8*, a G-protein-coupled receptor that inhibits cell migration upon binding *S*-geranylgeranyl-l-glutathione, which is present in bile salts^29^; another subset expressed *CYSLTR1*, which binds to another glutathione-conjugated lipid mediator, LTC4^29^. T_FH_ expressing high levels of *CYSLTR1* were not previously identified in lymph nodes and may be specific to IEL^30^. These T_FH_ subsets showed other distinctive markers: P2RY8^+^ T_FH_ expressed *CXCR5* and *TNFSF8* (for CD30L); CysLTR1^+^ T_FH_ expressed *BTLA* and *CD200. P2RY8* mediates the retention of T_FH_ and B cells in the germinal center, while BTLA restrain T_FH_ germinal center responses^29^. *TNFSF8* gene polymorphisms have been associated with risk of CD^44^. Although IEL T_FH_ did not show mRNA for the B cell stimulatory cytokine IL-21, P2RY8^+^, and CysLTR1^+^ T_FH_ subsets may control mucosal B cell responses through other mechanisms, such as BTLA–HVEM and TNFSF8–TNFRSF8 interactions.

IEL CD8^+^ T cells were also quite heterogeneous, including canonical CD103^–^KLRG1^+^ cytotoxic CD8^+^ T cells and multiple subsets of tissue-resident CD8^+^ T cells, which shared the expression of CD103 and CD160 that may secure retention of T cells through binding to E-cadherin and HVEM on epithelial cells, respectively. Moreover, CD160 may shape the function of CD8^+^ T cells by inducing IFN-γ^45^. Tissue-resident CD8^+^ T cells included a subset of IL-7R^+^TCF7^+^ cells producing the DC chemoattractants XCL1 and XCL2^26^, which may recruit DCs from the LP. Other resident CD8^+^ T cells subsets were prone to effector functions: one subset expressed IFN-γ and the NK receptor KLRC1 specific for HLA-E; another expressed cytotoxicity mediators, lacked NK receptors but expressed the membrane protein EMP3, which has yet unknown function in immune responses. Finally, a group of resident CD8^+^ T cells expressed *EIF5A, MDM4*, and *SET*, which control p53 activity^28^, suggesting evasion from apoptosis and senescence. Many tissue-resident CD8^+^ T cells expressed S100A family members, which may contribute to antimicrobial functions.

One remarkable result of this study is the identification of at least two subsets of γδ T cells within the IEL. One subset expressed T_H_17 markers, such as *RORC*, *IL-23R*, *IL-22*, and *IL-26*. RORγt^+^ γδ T cells have been extensively described in mouse^47,48^ but not in human. Distinctive features of these γδ T cells included the expression of TCRVδ1, CD39, and NKp30, an activating cell surface receptor specific for B7H6^49^. Importantly, NKp30^+^ γδ T cells expressed higher levels of RORγt ex vivo as compared to NKp30^–^ γδ T cells and engagement of NKp30 resulted in IL-26 production, suggesting that these cells may have a protective function during homeostasis. Some traits of these γδ T cells, such as NKp30 expression and IL-26 production, were recently reported in a subset of CD8 T cells expanded in colon of UC patients^10^. Whether these colonic cells are bona fide CD8 T cells or γδ T cells expressing CD8, as we find in the small intestine, remains to be established. Another γδ T cell subset expressed the canonical γδ T cell transcription factor *ID3*^32^; this subset expressed NK cell receptors and produced *CSF1* and *PDGFD*, pointing to a potential crosstalk with macrophages and epithelial cells. While subsets of human γδ secreting *CSF1* have been reported^46^, *PDGFD* secretion by γδ T cells has not been described before. In addition, a rare cell subset expressing γ-constant region of γδTCR and *CD8A* expressed *FOXP3*. Future studies will be required to precisely identify these cells and to test their regulatory function.

Notably, our cohort of severe adult CD showed a reduction of IEL CD8^+^ T cells and γδ T cells. Consistently, a population of CD39^+^ CD8^+^ T cells and γδ T cells has been recently reported to decrease in colonic mucosa biopsies of pediatric CD patients^9^. This population overlaps with the γδ and CD8^+^ T cell subsets expressing NK cell receptors and CD39 reported here. While we observed a global reduction rather than a selective loss of specific subsets of CD8 and γδ T cells, this discrepancy may depend on differences in patient ages (children vs. adults), sampling location (colon vs. ileum), type of specimen (biopsies vs. surgical resections) or degree of disease.

Finally, analysis of LP T cell transcriptomes identified unique functional features not immediately related to the classical T cell functional modules. A subset of LP CD4^+^ T cells expressed heat-shock induced stress-pathway genes and cytokines, such as TNF and IL-2. A T cell subset expressing heat-shock proteins was also among five IEL populations recently reported in CD^50^. Intriguingly, a recent study showed that febrile temperature in mice induces T_H_17 differentiation and augments pathogenicity through heat-shock response genes^51^. Together, these observations highlight the involvement of heat-shock response in the differentiation of T cells in the intestine. Expression of *GPX1* in Tregs of the LP revealed the activation of anti-oxidative pathways in intestinal T cells, which may be particularly relevant to CD, as *GPX1* gene polymorphisms have been associated with risk of CD^52,53^. These results will prompt future studies to determine the impact of these unique functions in CD pathogenesis.

## Methods

### Preparation of single-cell suspension from intestinal samples

Single-cell suspension was prepared as previously described^54^. Briefly, mucosal tissue from terminal ileum was separated from the muscular layer and serosa and cut into small pieces. Intraepithelial lymphocyte cells were extracted by rotating the tissue at room temperature for 40 min in Hank’s balanced salt solution, 10% FCS, and 5 mM ethylenediaminetetraacetic acid (EDTA). Cells were filtered through 100-μm cell strainers and dithiothreitol (DTT) was added at a final concentration of 5 mM. After intraepithelial lymphocyte removal, LP cells were extracted by digesting tissue in complete RPMI medium containing 1 mg ml^–1^ Collagenase IV (Sigma, C-5138) at 37 °C for 1 h under agitation. Cells were filtered and subjected to density gradient centrifugation using 40 and 70% Percoll solutions. Cells were collected, sorted, and processed for scRNA-seq or collected and stained for CyTOF. From a set of patients cells were processed and frozen for later CyTOF or flow cytometry analysis. Control patients for the present study were patients undergoing abdominal surgery for colon cancer or polyposis, which had non-involved terminal ileum removed, as part of the surgical procedure.

All human studies were conducted under the approval of the Institutional Review Boards of Washington University. All ileum samples were provided as surgical waste with no identifiers attached on written informed consent to the Digestive Disease Research Cores Center at Washington University. The demographic data provided in this study will not allow patient identification.

### Immunohistochemistry

Formalin-fixed paraffin-embedded tissue blocks used for this study were retrieved from the tissue bank of the Department of Pathology (ASST, Spedali Civili di Brescia, Brescia, Italy). Four-micron thick tissue sections were used for immunohistochemical staining. Sections were incubated with anti-human CD4 (clone 4B12 1:50 Thermo Scientific) and antihuman CD8 antibody (clone C8-144B Agilent 1:50) and the reaction was revealed using Novolink Polymer (Leica Microsistem). For double staining, after completing the first immune reaction, the second was visualized using Mach 4 MR-AP (Biocare Medical), followed by Ferangi Blue. Finally, the slides were counterstained with Meyer’s Haematoxylin.

### Antibodies

Information on the antibodies used for flow cytometry and sorting is available in Supplementary Data 8.

### Flow cytometry and sorting

Cells were sorted on BD FACS Aria II and flow cytometry analyses were performed on BD Symphony A3 instrument. Data were analyzed by FlowJo software v10.7.1 (TreeStar).

### scRNA-seq and data analysis

T cells were sorted from processed IEL and LP as CD45^+^, lymphocyte gate, singlets, and alive CD3^+^ CD19^–^ expression. Sorted cells were sequenced using 10X Genomics platform with chemistry version 2. Cell Ranger pipeline (https://support.10xgenomics.com/single-cell-gene-expression/software/over-view/welcome) was used to process Chromium single-cell RNA-seq output to align reads and generate gene-cell expression matrices. Briefly, short sequencing reads were aligned to the GRCh38 reference genome and Ensembl^55^ transcriptome by STAR^56^. The uniquely aligned reads were used to quantify gene expression levels for all Ensembl genes. We filtered out low-quality cells from the dataset if the number of genes detected was <500 or >3000, or the percentage of mitochondrion reads was >15%. Mitochondrion and ribosomal genes usually consumed a large fraction of reads in our dataset, and their relative abundance varied significantly from sample to sample. Such genes were not interesting in our research, and thus were excluded for downstream data analysis. Additionally, all genes that were not detected in at least 1% of all our single cells were discarded. Average UMI were 2960 and 3020 for IEL and LP T cells, respectively.

#### scRNA-seq downstream analysis

Downstream analyses were performed using Seurat R software package version 3.0 (http://satijalab.org/seurat/). After removing unwanted cells and genes from the dataset, raw UMIs in each cell were first scaled by library size and then log-transformed. To improve downstream dimensionality reduction and clustering, we first regressed out unwanted source of variation arising from the number of detected molecules. Then highly variable genes were identified and selected for PCA reduction of high-dimensional data. Cells in this reduced spaced were harmonized to adjust for batch effects coming from multiple donors including both normal and CD using the Harmony tool implemented in Seurat v3^57^. These low dimensional corrected Harmony embeddings were used for downstream analyses. Graph-based clustering was performed on the reduced data for clustering analysis with Seurat v3. The resolution in the FindClusters function in Seurat was set to 0.6 and the clustering results were shown in a UMAP plot. For different cell types, cells were grouped based on top markers. MAST in Seurat v3 was used to perform differential analysis^58^. For each cluster, DEGs were generated relative to all of the other cells.

### CyTOF acquisition and analysis

All antibodies information is available in Supplementary Data 9. Cells were washed with Cy-FACS buffer (CyPBS, Rockland, MB-008; 0.1% BSA, Sigma, A3059; 0.02% Sodium Azide, Sigma, 71289, 2 mM EDTA, Hoefer, GR123-100) stained on ice for an hour. After two washes cells were stained with cisplatin (Enzo Life Sciences, NC0503617) for 1 min, washed again twice, fixed in 4% PFA (Electron Microscopy Sciences, 15710) for 15 min, spun down and re-suspended in Intercalator-Ir125 (Fluidigm, 201192 A) overnight. Cells were washed and counted and analyzed on a CyTOF 2 mass cytometer (Fluidigm). Samples were manually gated using Cytobank. Background, dead cells (Cisplatin^+^), doublets (DNA1/2 stain), and normalization beads were excluded. Dimensionality reduction analysis was performed by equally sampling CD45^+^ CD3^+^ CD19^−^ cells per donor based on 20 different markers (CD4, CD8a, NKp44, CD127, CD45RA, CD103, TIGIT, CCR4, CD39, CD45RO, CCR6, CD25, TCRγδ, CD161, NKG2A, CD226, CD94, CD56, CD27, and CD294) using the viSNE tool^59^ in Cytobank to apply the Barnes–Hut implementation of the t-SNE algorithm. viSNE data were exported from Cytobank and uploaded into MATLAB implementation of Phenograph^60^ and transformed using a cofactor of five for subsequent clustering analysis. Visualization of clusters identified by Phenograph was done using the R package ggplot2. CITRUS analysis was performed by equally sampling CD45^+^ CD3^+^ CD19^−^ CD4^+^ (1807) cells per sample based on the expression of 14 markers (CD45RO, CD45RA, CCR6, CCR4, CD161, CD127, CD25, CD39, TIGIT, CD117, CD103, CD27, NKp44, and CD226) using the CITRUS tool in Cytobank. Significant changes in cell frequency were inferred with SAM (a nonparametric correlative method) for an FDR <1%. Every cluster displayed contains at least 3% of all clustered cells and are scaled on the basis of frequency of cells in each cluster.

### Ex vivo intracellular staining of γδ T cells for RORγt

Total γδ T cells were sorted from IEL of control patients and cell surface stained for NKp30, CD39, fixed and permeabilized and stained intracellularly for RORγt with eBioscience FoxP3 staining kit.

### In vitro T cell culture and NKp30 crosslinking

NKp30^+^ γδ T cells were sorted from IEL of control patients and expanded in vitro with PHA (HA16, Remel), irradiated feeder and IL-2. Expanded cells were stimulated with plate bound anti-NKp30 (clone 30.95.1) or isotype control (CRL-1729, ATCC). IL-26 was measured in supernatants 72 h later by ELISA (CUSABIO).

### Statistical Analysis

Statistical analysis was performed using Graphpad Prism 8.4.3 (GraphPad Software, La Jolla, CA) or R version 3.6.2 (2019), as indicated in the figure legends. Data were presented as median and interquartile range. Unless otherwise noted, statistically significant differences between groups were determined by ordinary one-way ANOVA. In all figures, the following symbols were used to designate significance: *P ≦ 0.05, **P  ≦ 0.01, ***P ≦ 0.001.

### Reporting Summary

Further information on research design is available in the Nature Research Reporting Summary linked to this article.

## Supplementary information

Supplementary Information

Reporting Summary

Description of Additional Supplementary Files

Supplementary Data 1

Supplementary Data 2

Supplementary Data 3

Supplementary Data 4

Supplementary Data 5

Supplementary Data 6

Supplementary Data 7

Supplementary Data 8

Supplementary Data 9

## Data Availability

The scRNA-seq have been deposited in GEO under the GSE157477. Source data are provided with this paper.

## Source data

Source Data
