# Supplementary material for: Single-cell analyses of Crohn’s disease tissues reveal intestinal intraepithelial T cells heterogeneity and altered subset distributions
Source: Nat Commun. 2021 Mar 26;12:1921. doi: 10.1038/s41467-021-22164-6 (PMC7997960; doi:10.1038/s41467-021-22164-6)
Supplement: Supplementary file 1 — Supplementary Information [file 41467_2021_22164_MOESM1_ESM.pdf]

# Single-cell analyses of Crohn's disease tissues reveal intestinal intraepithelial T cells heterogeneity and altered subset distributions

Natalia Jaeger, Marco Colonna

**Supplementary Figure 1**

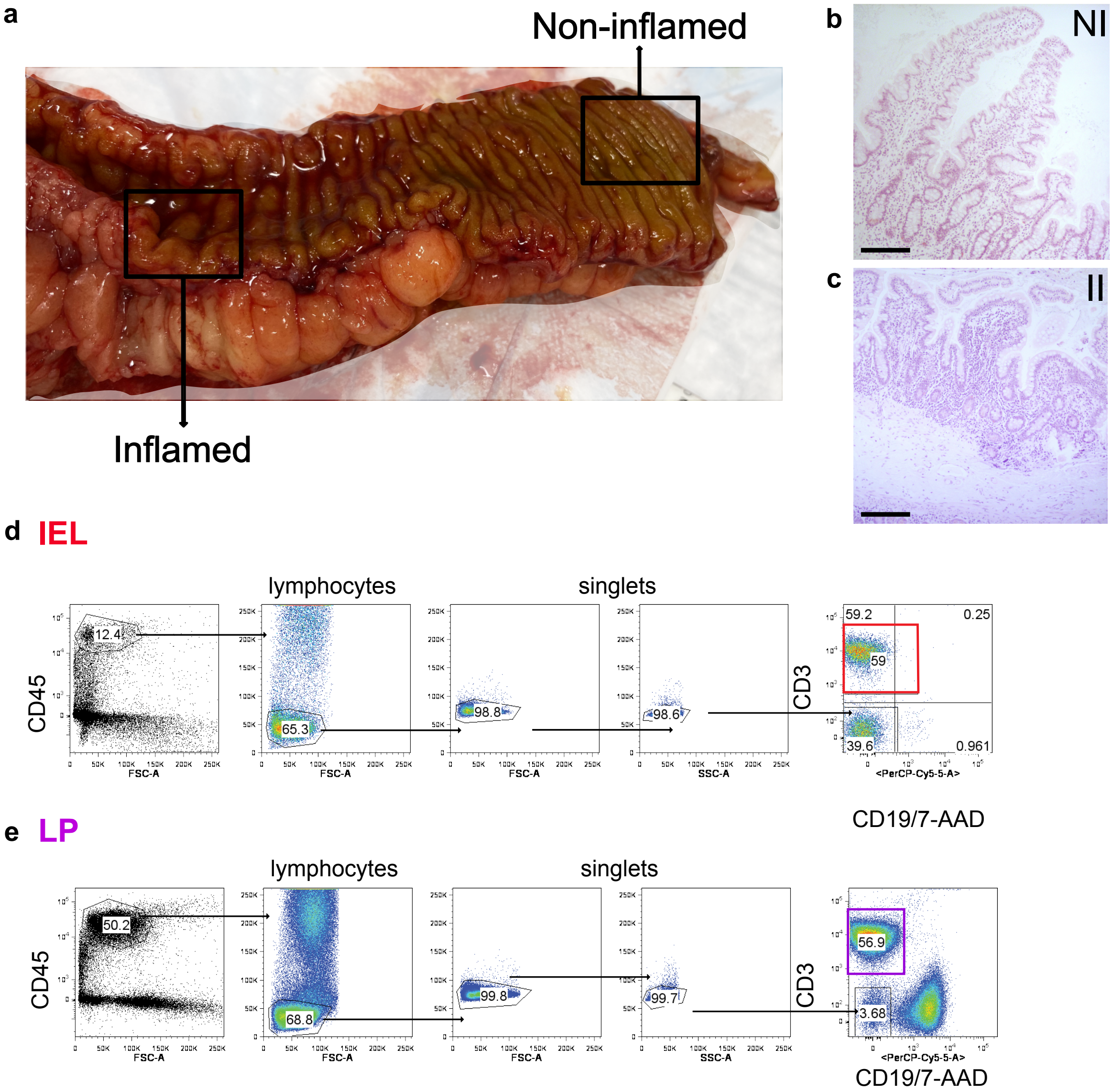


**Supplementary Figure 1. Tissue collection from paired NI and II areas and gating strategy for T cell sorting from IEL and LPL. a**, Picture showing the paired NI and II areas collected from surgically resected terminal ileum from CD patients. **b-c**, Hematoxylin and eosin staining of terminal ileum samples from paired NI (**b**) and II (**c**) areas. This experiment was performed once. Magnification: 20x, scale bar: 100 µm. **d-e**, Gating strategy to sort T cells used on scRNA-seq data shown in Figure 1, 4, 5, Supplementary Figure 2, 4, 5, 6. Gating was applied on alive $CD45^+$, lymphocytes, singlets, $CD3^+CD19^-$ cells.

**Supplementary Figure 2**

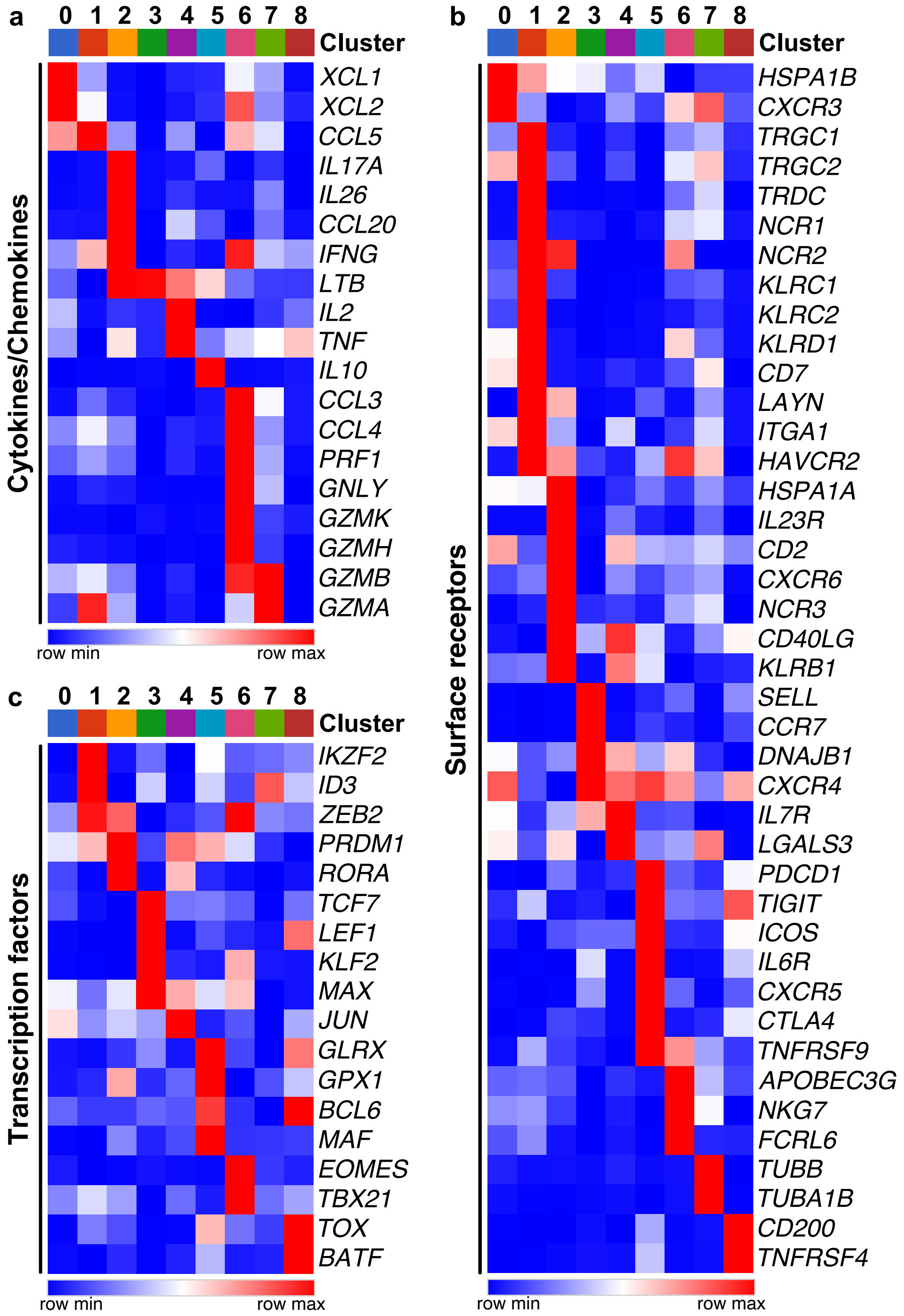


**Supplementary Figure 2. Expression of transcription factors, cell surface receptors and soluble factors in each IEL T cell cluster. a**, Heat map depicting the expression level of selected cytokines, chemokines and cytotoxicity mediators in clusters 0 to 8. **b**, Heat map depicting the expression level of selected cell surface receptors in clusters 0 to 8. **c**, Heat map depicting the expression level of selected transcription factors in clusters 0 to 8.

## Supplementary Figure 3

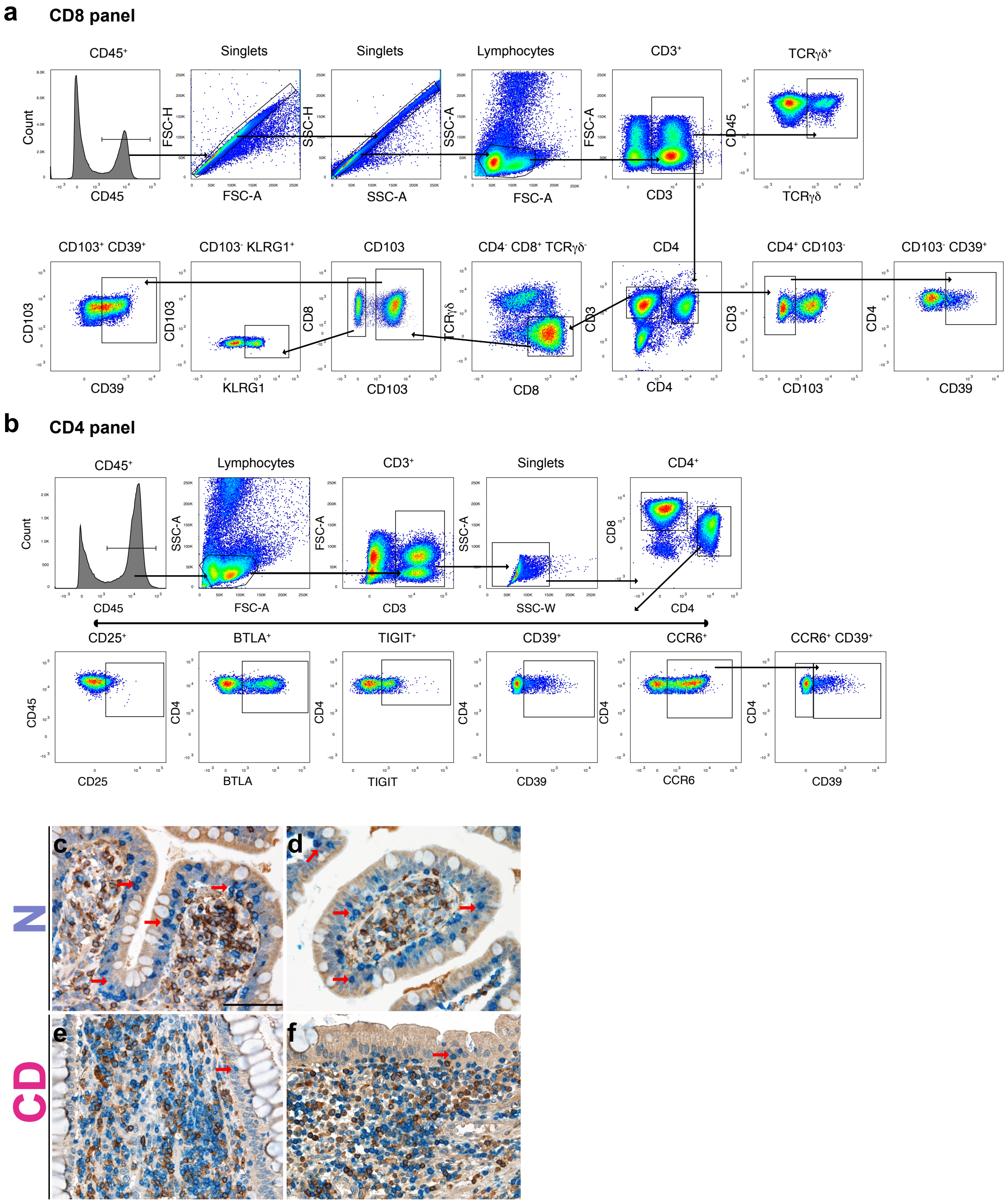


**Supplementary Figure 3. Gating strategy to quantitate IEL cell subsets by flow cytometry**. Gating strategy used to quantify by flow cytometry the different IEL CD8$^+$, TCRγδ$^+$ and CD4$^+$ T cell subsets in terminal ileum of controls and CD patients. T cells were gated as singlets, CD45$^+$CD3$^+$. Gating strategy for the CD4 (**a**) and CD8 (**b**) panel presented on Figures 2 and 3. Gating is shown on the top of each panel. **c-f,** IHC of tissue sections stained with anti-CD4 and CD8 from terminal ileum sections of control (**c, d**) and CD (**e, f**) patients. Images are representative of two control (**c, d**) and three CD (**e, f**) patients. Sections are counterstained with hematoxylin. Red arrows indicate CD8$^+$ T cells in the IEL. Magnification: 200x, scale bar: 100 μm. N= control, CD = Crohn's disease.

## Supplementary Figure 4

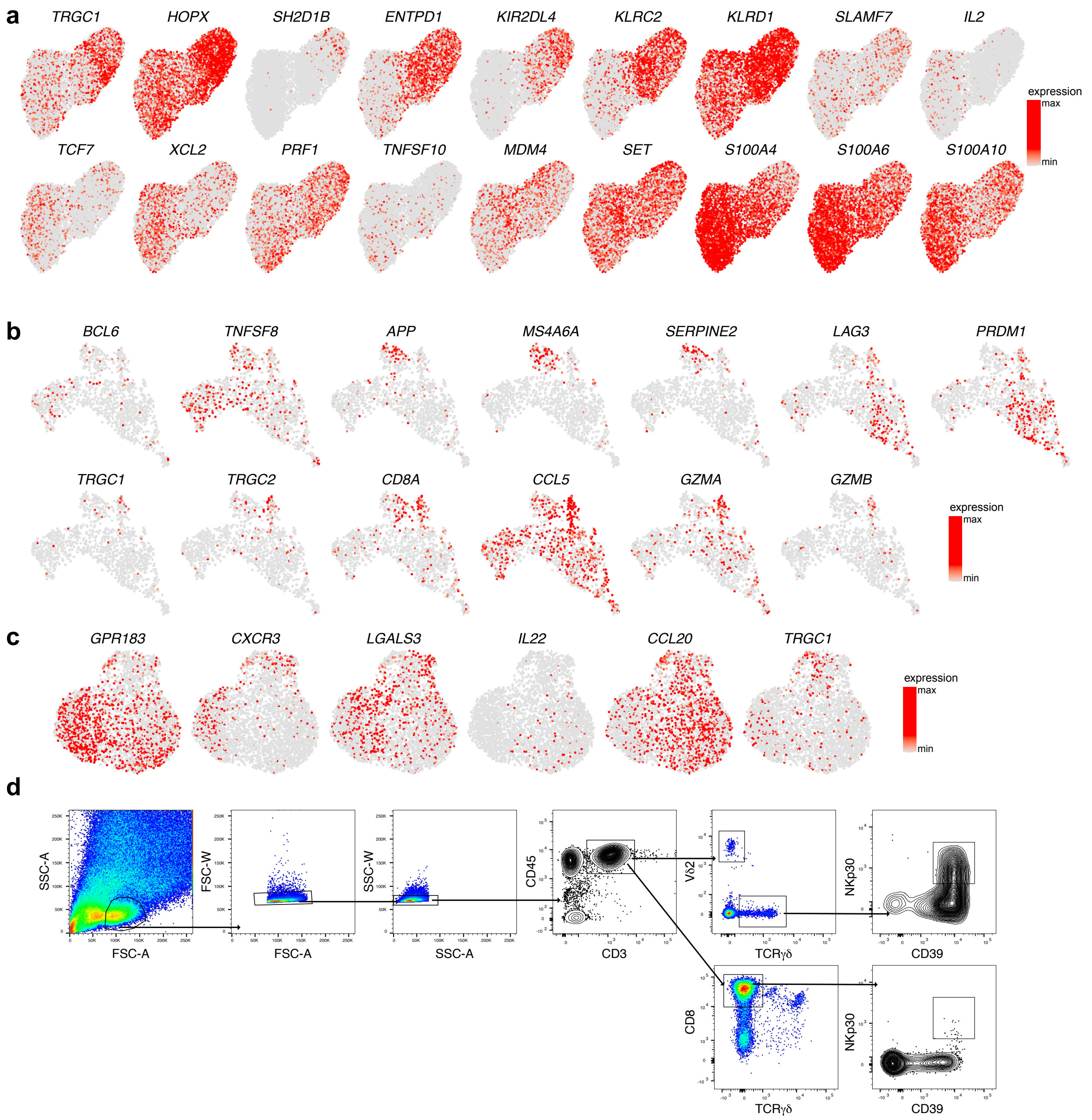


**Supplementary Figure 4. Reclustering of heterogenous IEL CD8⁺ and CD4⁺ T cells populations. a,** UMAP of representative selected genes associated with the reclustering of clusters 0 and 1 from Fig. 1a. **b,** UMAP of representative selected genes associated with the reclustering of clusters 5 and 8 from Fig. 1a. **c,** UMAP of representative selected genes associated with the reclustering of clusters 2 and 4 from Fig. 1a. **d,** Gating strategy for IEL γδ and CD8 T cell expressing NKp30 of the terminal ileum of controls patients. Data presented on Figure 4j-i . Gating was applied on lymphocytes, singlets, $CD45^+$, $CD3^+$ cells and $TCR\gamma\delta^+$ or $V\delta2^-$ for $\gamma\delta^+$ T cells or $CD8^+$ $TCR\gamma\delta^+$ for CD8 T cells.

**Supplementary Figure 5**

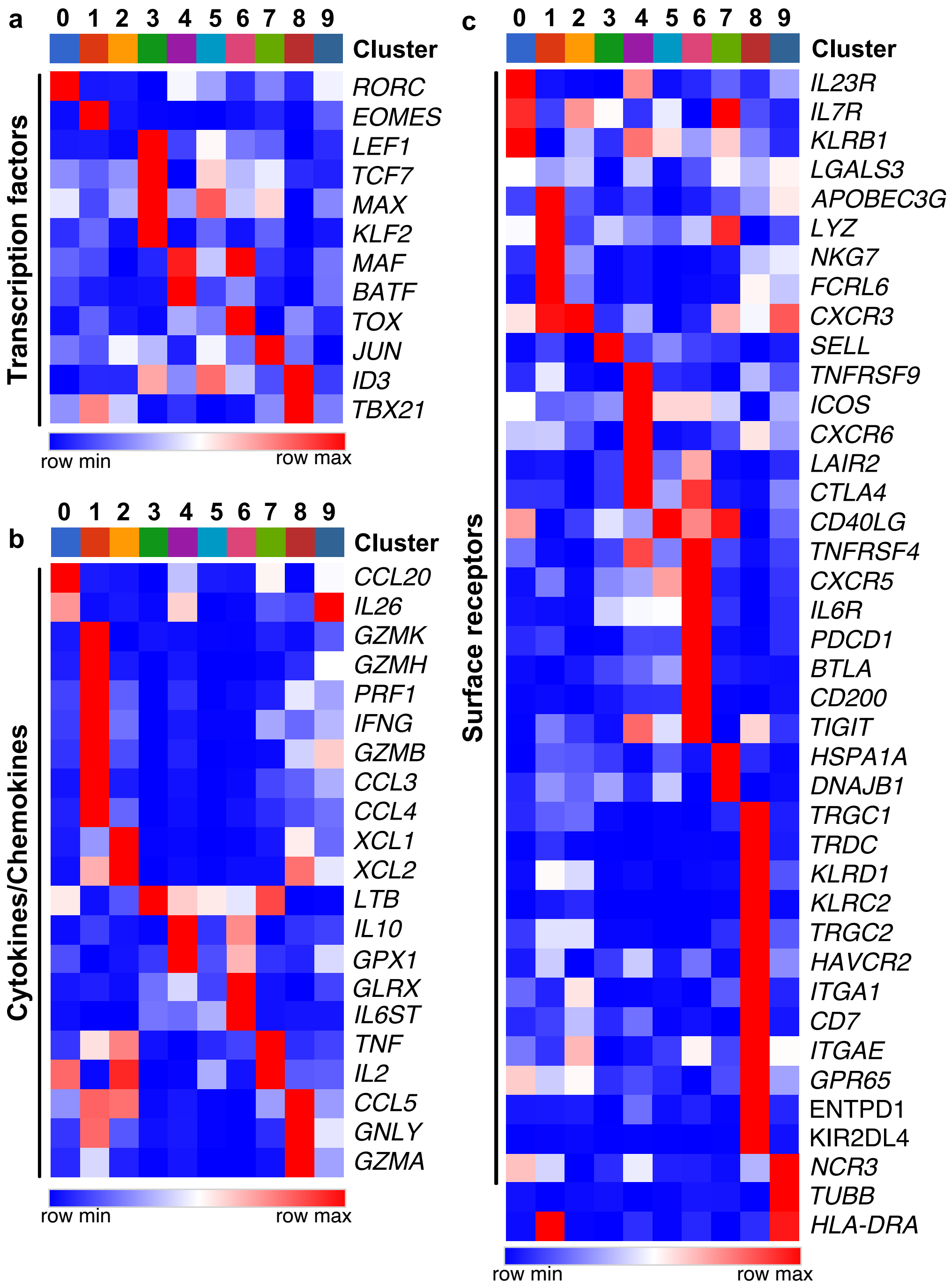


**Supplementary Figure 5. Expression of transcription factors, cell surface receptors and soluble factors in each LP T cell cluster. a**, Heat map depicting the expression level of selected transcription factors in clusters 0 to 9. **b**, Heat map depicting the expression level of selected cytokines, chemokines and cytotoxicity mediators in clusters 0 to 9. **c**, Heat map depicting the expression level of selected cell surface receptors in clusters 0 to 9.

**Supplementary Figure 6**

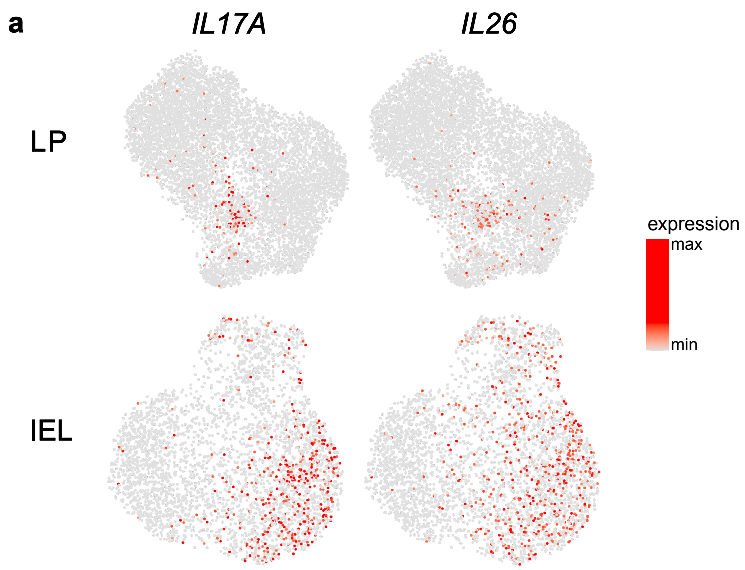


**Supplementary Figure 6. IEL contains more activated Th17 cells. a,** UMAP plots depicting the expression of *IL17A* and *IL26* in the LP and IEL reclustering of clusters 0 (LP, Fig. 5a) and 2 and 4 (IEL, Fig.1a).

# Supplementary Figure 7

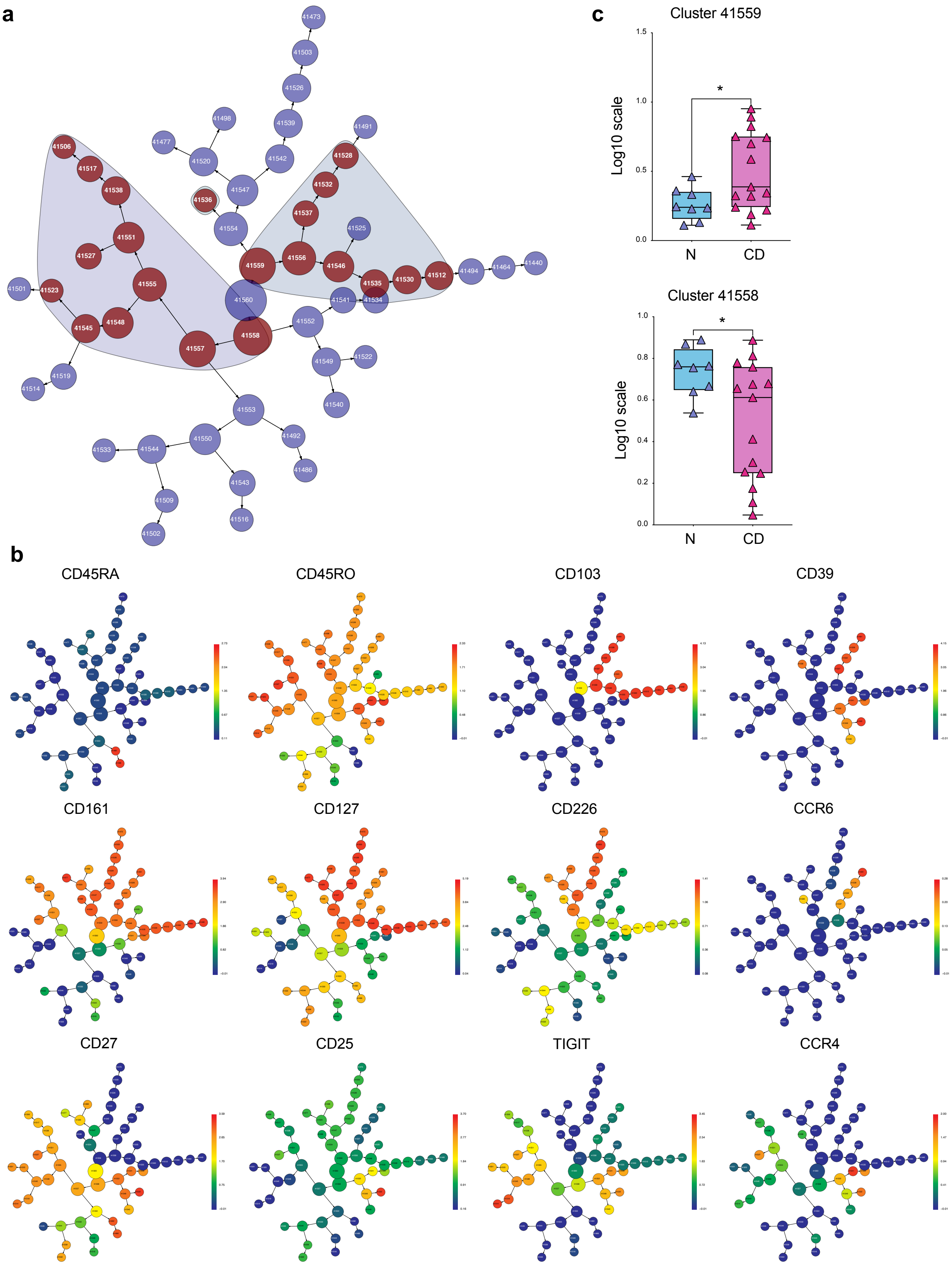

**Supplementary Figure 7. Hierarchical clustering analysis of CD4 T cells subsets by Citrus. a**, Schematic CITRUS plot generated to identify and quantify $CD4^+$ T cells subsets from normal and CD lamina propria samples. **b**, Expression markers that identify the nodes in $CD4^+$ T cells. **c**, Frequencies of cluster 41559 (Th17) and cluster 41558 ($T_{FH}$) (normal vs CD). Triangles on the boxplots show data collected for each individual donor. Data are median and interquartile range. Significance was calculated using a t-student test with Prism v8 software. **c-d**, *$P=0.0319$.* **a-d**, Controls (N), n=8; CD (combined inflamed (**I**I) and non-inflamed (NI) samples), n=15 (9 NI, 6 **I**I, total of 10 different patients). Samples were fresh at the time of staining.
