# Supplementary material for: Single-cell analyses of Crohn’s disease tissues reveal intestinal intraepithelial T cells heterogeneity and altered subset distributions
Source: Nat Commun. 2021 Mar 26;12:1921. doi: 10.1038/s41467-021-22164-6 (PMC7997960; doi:10.1038/s41467-021-22164-6)
Supplement: Supplementary file 2 — Reporting Summary [file 41467_2021_22164_MOESM2_ESM.pdf]

nature research

Corresponding author(s): Scott A. Jelinsky and Marco Colonna

Last updated by author(s): Feb 27, 2021

Reporting Summary

Nature Research wishes to improve the reproducibility of the work that we publish. This form provides structure for consistency and transparency in reporting. For further information on Nature Research policies, see our Editorial Policies and the Editorial Policy Checklist.

Statistics

For all statistical analyses, confirm that the following items are present in the figure legend, table legend, main text, or Methods section.

n/a	Confirmed	
☐	☒	The exact sample size (*n*) for each experimental group/condition, given as a discrete number and unit of measurement
☐	☒	A statement on whether measurements were taken from distinct samples or whether the same sample was measured repeatedly
☐	☒	The statistical test(s) used AND whether they are one- or two-sided<br>*Only common tests should be described solely by name; describe more complex techniques in the Methods section.*
☐	☒	A description of all covariates tested
☐	☒	A description of any assumptions or corrections, such as tests of normality and adjustment for multiple comparisons
☐	☒	A full description of the statistical parameters including central tendency (e.g. means) or other basic estimates (e.g. regression coefficient) AND variation (e.g. standard deviation) or associated estimates of uncertainty (e.g. confidence intervals)
☐	☒	For null hypothesis testing, the test statistic (e.g. *F*, *t*, *r*) with confidence intervals, effect sizes, degrees of freedom and *P* value noted<br>*Give P values as exact values whenever suitable.*
☒	☐	For Bayesian analysis, information on the choice of priors and Markov chain Monte Carlo settings
☒	☐	For hierarchical and complex designs, identification of the appropriate level for tests and full reporting of outcomes
☒	☐	Estimates of effect sizes (e.g. Cohen's *d*, Pearson's *r*), indicating how they were calculated

Our web collection on statistics for biologists contains articles on many of the points above.

Software and code

Policy information about availability of computer code

Data collection	Flow cytometry (FACS) - BD FACS Diva Software v8.0.1 (BD FACS Aria II, BD FACSymphony machine, BD FACS LSRFortessa and BD FACS LSRFortessa X20 machine)<br>Cytometry Time of Flight (CyTOF) Data acquisition - Fluidigm CyTOF Software v6.7 (CyTOF2 Helios machine)
Data analysis	Cell Ranger pipeline (https://support.10xgenomics.com /single-cell-gene-expression/software /over-view/welcome) was used to process Chromium single-cell RNA-seq output to align reads and generate gene-cell expression matrices. Briefly, short sequencing reads were aligned to the GRCh38 reference genome and Ensembl 41 transcriptome by STAR 42. The uniquely aligned reads were used to quantify gene expression levels for all Ensembl genes. Downstream analyses were performed by R software package Seurat (http://satijalab.org/ seurat/). ScRNA-seq downstream analysis. Downstream analyses were performed by R software package Seurat (http://satijalab.org/ seurat/). Raw reads in each cell were first scaled by library size and then log-transformed. To improve downstream dimensionality reduction and clustering, we first regressed out unwanted source of variation arising from the number of detected molecules. Then highly variable genes were identified and selected for PCA reduction of high-dimensional data. Based on elbow plot, the top 25 PCs were selected for unsupervised clustering of cells. The resolution in the FindClusters function in Seurat was set to 0.6 and the clustering results were shown in a UMAP plot. Accordingly, the marker genes in each cluster were identified by t-test implemented in the Seurat v2.3 package. Harmonization of gut samples from merged donors was performed as described (https://doi.org/10.1101/461954).<br><br>CyTOF analysis: Dimensionality reduction analysis was performed by equally sampling CD45+ CD3+ CD19- cells per donor based on 20 different markers using the viSNE tool in Cytobank to apply the Barnes-Hut implementation of the t-SNE algorithm. viSNE data were exported

from Cytobank and uploaded into MATLAB (R2019b Update 1) implementation of Phenograph (Cyt (SightOf) V2) and transformed using a cofactor of five for subsequent clustering analysis. Visualization of clusters identified by Phenograph was done using the R package ggplot2 (3.3.0). Citrus analysis was performed in Cytobank

FACS data were analyzed by FlowJo software v10.7.1 (TreeStar).

Statistical analysis was performed using Graphpad Prism 8.4.3 (GraphPad Software, La Jolla, CA) or R version 3.6.2 (2019).

For manuscripts utilizing custom algorithms or software that are central to the research but not yet described in published literature, software must be made available to editors and reviewers. We strongly encourage code deposition in a community repository (e.g. GitHub). See the Nature Research guidelines for submitting code & software for further information.

Data

Policy information about availability of data

All manuscripts must include a data availability statement. This statement should provide the following information, where applicable:

- Accession codes, unique identifiers, or web links for publicly available datasets
- A list of figures that have associated raw data
- A description of any restrictions on data availability

The scRNAseq have been deposited in GEO under the GSE157477 (https://www.ncbi.nlm.nih.gov/geo/query/acc.cgi?acc=GSE157477). The raw numbers for charts and graphs are available in the Source Data file whenever possible.

Field-specific reporting

Please select the one below that is the best fit for your research. If you are not sure, read the appropriate sections before making your selection.

☒ Life sciences ☐ Behavioural & social sciences ☐ Ecological, evolutionary & environmental sciences

For a reference copy of the document with all sections, see nature.com/documents/nr-reporting-summary-flat.pdf

Life sciences study design

All studies must disclose on these points even when the disclosure is negative.

Sample size

Sample size was determined based on the complexity, cost of the experiments and sample availability. For Flow Cytometry and CyTOF, sample size was based on tissue availability during the course of the study.
For IEL FACS data: IEL CD4/CD8 T cell quantification (frozen and fresh samples): control (N), n=33, CD, non-inflamed (NI) n=19, CD inflamed site (II), n=19. γd T cell quantification (frozen and fresh samples): control (N) n=34, CD, non-inflamed site (NI), n=20; CD inflamed site (II), n=19. Remaining data (frozen samples): control (N), n=15, CD (NI and II), n=9, each.
LP CD4 and CD8 quantification (by CyTOF + FACS) (frozen and fresh samples): control (N), n=17; CD, non-inflamed site (NI) n=19; CD, inflamed site (II), n=14. Total of 38 individual patients.
For CyTOF data alone (fresh samples): control (N), n=8; CD, non-inflamed site (NI) n=9; CD, inflamed site (II), n=6. Total of 18 individual patients.
For LP FACS data alone (frozen samples): control (N), n=9; CD, non-inflamed site (NI) n=10; CD, inflamed site (II), n=8. Total of 20 individual patients.
For scRNAseq data 2 N, 2 CD (both non-inflamed and inflamed) patients were analyzed.

Data exclusions

Data were excluded from CyTOF analysis when the technical quality was not satisfactory (e.g. Barium contamination, low number of events).

Replication

The number of experiments performed for each panel is indicated in Figure legend. Data were replicated with independent cohort of patients.

Randomization

Randomization was not performed for this study. Covariates are reported in the Supplementary Tables but were not analyzed.

Blinding

Blinding was not required for this study because it was critical to identify if samples were control or CD patient to interpret the data.

Reporting for specific materials, systems and methods

We require information from authors about some types of materials, experimental systems and methods used in many studies. Here, indicate whether each material, system or method listed is relevant to your study. If you are not sure if a list item applies to your research, read the appropriate section before selecting a response.

Materials & experimental systems

n/a	Involved in the study	
☐	☒	Antibodies
☒	☐	Eukaryotic cell lines
☒	☐	Palaeontology and archaeology
☒	☐	Animals and other organisms
☐	☒	Human research participants
☒	☐	Clinical data
☒	☐	Dual use research of concern

Methods

n/a	Involved in the study	
☒	☐	ChIP-seq
☐	☒	Flow cytometry
☒	☐	MRI-based neuroimaging

Antibodies

Antibodies used

Flow Cytometry (dilution 1:50, unless otherwise noted).
Biolegend: CD161-BV510 (339922); TIGIT-BV605 (372711); CD39-FITC (328206), CD19-PerCP (302230), GP130-PE (362003), BTLA-APC-Cy7 (344517), ICOS-APC (313509), CCR7-BV785 (353229), CD25-AF700 (302621), CD103-BV421 (350213), CD160-APC (341207), CXCR3-biotin (353743), NKp30-PE (325208), CD45-AF700 (368514), TCRgd-PE-Cy7 (331221), CD3-BV510 (300447), Vd2-BV421 (331427), CD3-BV605 (317322), PD-1-BV421 (329920), Streptavidin-PE-Cy7 (405206) (1:100).
eBioscience™: CD45 APC (17-0459-42), CD8-PerCP-Cy5.5 (45-0088-42), TCRgd-PE (12-9959-42), KLRG1-eFluor710 (46-9488-42), RORgt-APC (17-6988-82)
BD: CD4-BUV737 (612761), CD45-BUV395 (563791), CD3-BUV661 (565066), CCR6-Biotin (559561), 7-AAD (559925) (1:200).
More information available in Supplementary Table 8.

CyTOF: NKp44-167Er (eBioscience, 16-3369-85). Fluidigm: CD45-089Y (3089003B); CD196-141Pr (3141003A); CD19-142Nd (3142001B); CD117-143Nd (3143001B); CD4-145Nd (3145001B); CD8a-146Nd (3146001B); CD25-149Sm (3149010B); FceR-150Nd (3150027B); CD138-150Nd (3150012B); CD103-151Eu (3151011B); TCRgd-152Sm (3152008B); TIGIT-153Eu (3153019B); CD3-154Sm (3154003B); CD85j-156Gd (3156020B); CD194-158Gd (3158032A); CD161-159Tb (3159004B); CD39-160Gd (3160004B); CD27-162Dy (3162009B); CD45RO-165Ho (3165011B); CD34-166Er (3166012B); CD127-168Er (3168017B); CD159-169Tm (3169013B); CD45RA-170Er(3170010B); CD226-171Yb (3171013B); CD354(TREM1)-172Yb (3172022B); CD94-174Yb (3174015B); CD14-175Lu (3175015B); CD56-176Yb (3176009B) and CD16-209Bi (3209002B). CD303-147Sm (3147009B); CD1b-155Gd (3155007B); DR3-161Dy (3161003B); CD294-163Dy (3163003B); CD141-173Yb (3173002B). Dilution for each antibody used in the CyTOF panel is shown in Supplementary Table 9

Validation

All antibodies were bought from commercial suppliers and are validated by the vendor for the species and assay used in our study. Validation data is available on the vendors' websites. All antibodies for CyTOF were initially tested against unstained controls and titration was performed to optimize. Validation was also confirmed by the authors by linking RNA expression with protein data.

Human research participants

Policy information about studies involving human research participants

Population characteristics

Gut specimens from control and Crohn's Disease patients were obtained as surgical waste upon IRB approval. Patient's demographics are presented in details on Supplementary Table 1 and 2.

Recruitment

Patients were recruited to this study provided their were able and gave their written informed consent to participate in the study.

Ethics oversight

All human studies were conducted under the approval of the Institutional Review Boards of Washington University. All ileum samples were provided as surgical waste with no identifiers attached on written informed consent to the Digestive Disease Research Cores Center at Washington University. IRB ID: 201106341

Note that full information on the approval of the study protocol must also be provided in the manuscript.

Flow Cytometry

Plots

Confirm that:

- ☒ The axis labels state the marker and fluorochrome used (e.g. CD4-FITC).
- ☒ The axis scales are clearly visible. Include numbers along axes only for bottom left plot of group (a 'group' is an analysis of identical markers).
- ☒ All plots are contour plots with outliers or pseudocolor plots.
- ☒ A numerical value for number of cells or percentage (with statistics) is provided.

Methodology

Sample preparation	Sample preparation is detailed in methods
Instrument	BD FACS LSR Fortessa, BD FACSymphony, BD FACS LSR Fortessa X20 were used for analysis. BD FACSAria II was used for sorting ad described in methods.
Software	Data were acquired on BD DIVA software and analyzed by Flowjo.
Cell population abundance	Cell sorting purity was between 98-99% in all experiments
Gating strategy	Sorting gating strategy: A gate was first applied on CD45+ cells to remove contaminating tissue cells. Lymphocytes by gating on FSC/SSC. Doublets were then removed by gating on FSC-W and SSC-W. T cells were gated on CD3+ CD19- cells. Gating strategy is illustrated in Supplementary Figure 1d (IEL) and 1e (LP).<br>FACS for t-SNE analysis: Gating strategy is illustrated in Supplementary Figure 3a (CD8 panel), 3b (CD4 panel).<br>FACS for NKp30 expression: Gating strategy is illustrated in Supplementary Figure 4d.

☒ Tick this box to confirm that a figure exemplifying the gating strategy is provided in the Supplementary Information.
