# Supplementary material for: Single-cell analyses of Crohn’s disease tissues reveal intestinal intraepithelial T cells heterogeneity and altered subset distributions
Source: Nat Commun. 2021 Mar 26;12:1921. doi: 10.1038/s41467-021-22164-6 (PMC7997960; doi:10.1038/s41467-021-22164-6)
Supplement: Supplementary file 3 — Description of Additional Supplementary Files [file 41467_2021_22164_MOESM3_ESM.pdf]

Description of Additional Supplementary Files:

Supplementary Data 1: Patient’s demographics.

Supplementary Data 2: Additional CD patient information.

Supplementary Data 3: Patient samples used in Figure 1, Supplementary Figure 2, Figure 4(a-g), Figure 5, Supplementary Figure 5, Supplementary Figure 6.

Supplementary Data 4: Patient samples used in Figure 2. NI = non-inflamed ileum, II = inflamed ileum.

Supplementary Data 5: Patient samples used in Figure 3. NI = non-inflamed ileum, II = inflamed ileum.

Supplementary Data 6: Patient samples used in Figure 4. NI = non-inflamed ileum, II = inflamed ileum. Samples were pooled before sorting (5 samples in each experiment).

Supplementary Data 7: Patient samples used in Figure 6 and Supplementary Figure 7. NI = non-inflamed ileum, II = inflamed ileum.

Supplementary Data 8: List of antibodies used for flow cytometry.

Supplementary Data 9: List of antibodies used for CyTOF.
